# PTEN restoration and CXCR2 depletion synergistically enhance the effect of enzalutamide and inhibit bone metastatic CRPC

**DOI:** 10.7150/thno.114534

**Published:** 2025-07-25

**Authors:** Jiyuan Chen, Luyao Gong, Simeng Cao, Guanshan Song, Yeheng Peng, Yuanyuan Wang, Yan-Ru Lou, Teemu J. Murtola, Yao Wu, Ganjun Yu, Yuan Gao

**Affiliations:** 1 School of Pharmaceutical Sciences, Fudan University, Shanghai 201203, China; 2 Department of Pharmacy, Shanghai Ninth People's Hospital, Shanghai Jiao Tong University, Shanghai 200011, China; 3 Shanghai Institute of Organic Chemistry, University of Chinese Academy of Sciences, Shanghai 200032, China; 4 TAYS Cancer Center, Department of Urology, Tampere University Hospital, Tampere 33100, Finland; 5 National Key Laboratory of Immunity and Inflammation & Institute of Immunology, College of Basic Medical Sciences, Naval Medical University, Shanghai 200433, China; 6 State Key Laboratory of Advanced Drug Formulations for Overcoming Delivery Barriers, Shanghai 201203, China

**Keywords:** bone metastatic CRPC, bone-targeting, lipid nanoparticles, nucleic acid delivery, enzalutamide

## Abstract

**Rationale:** Enzalutamide (Enz) is the first-line therapy for castration-resistant prostate cancer (CRPC). However, drug resistance has hindered its further application. Moreover, CRPC is frequently prone to metastasis, particularly to bone (BmCRPC).

**Methods:** To investigate the involvement of phosphatase and tensin homolog deleted on chromosome 10 (*PTEN*) deletion and C-X-C motif chemokine receptor 2 (CXCR2) overexpression in Enz-resistant CRPC and BmCRPC, we constructed a bisphosphonate (BP) lipid-like material with high bone affinity (GB4-BPL) for the codelivery of a PTEN plasmid (pPTEN) and CXCR2 siRNA (siCXCR2) to BmCRPC.

**Results:** GB4-BPL demonstrated twice the bone metastasis-targeting ability of GB4-lipo (which lacks bisphosphonate modification) while maintaining a gene transfection efficiency comparable to that of Lipo8000 and exhibiting significantly lower cytotoxicity. Moreover, siCXCR2 and pPTEN loaded in GB4-BPL (GB4-BPL@siCXCR2/pPTEN) synergistically inhibited tumor growth and metastasis, highly enhancing the effect of Enz by 69.45% in the Enz-resistant model. Furthermore, GB4-BPL@siCXCR2/pPTEN significantly reduced the numbers of MDSCs, Tregs, and M2-like TAMs by 55.01%, 64.75%, and 52.53%, respectively, while increasing the proportions of M1 macrophages, NK cells, and CD8^+^ T cells by 1.65-, 1.40-, and 4.60-fold, respectively. In addition, this nanosystem reduced skeletal-related events.

**Conclusions:** Our studies demonstrated the potential of GB4-BPL for delivering siCXCR2/pPTEN to tumor and bone metastatic sites. GB4-BPL@siCXCR2/pPTEN alone or in combination with Enz could provide a new strategy for the treatment of drug-resistant BmCRPC.

## Introduction

Prostate cancer (PCa) remains a leading cause of cancer-related death in men worldwide 1 and can progress to castration-resistant prostate cancer (CRPC) within 3 years of androgen deprivation therapy (ADT) 2,3. A substantial proportion of these patients with CRPC progress to metastatic CRPC (mCRPC), with nearly 90% of cases metastasizing to bone, leading to the development of bone metastatic CRPC (BmCRPC) 4. Enzalutamide (Enz), a second-generation androgen receptor (AR) inhibitor, is used as a first-line treatment for CRPC and mCRPC, prolonging patient survival 5,6. However, nearly all patients develop drug resistance, which leads to disease progression via complicated mechanisms 5. Previously, relevant strategies have been developed for the treatment of Enz-resistant PCa or bone metastatic PCa 3,6-8. However, they remain significant challenges in clinical translation. Therefore, the development of new strategies to overcome drug resistance and bone metastasis in PCa, especially BmCRPC, is urgently needed.

Genomic aberrations of phosphatase and tensin homolog deleted on chromosome 10 (*PTEN*), a tumor suppressor gene, negatively regulate the PI3K-AKT pathway, which is associated with primary (42%) and metastatic (~100%) PCa 9,10. It is estimated that more than 49% of patients with mCRPC have functional loss of *PTEN*, promoting resistance to AR-targeting therapy 11, partly because *PTEN* deletion promotes AR reactivation 12. Moreover, activation of Akt subsequent to *PTEN* loss is correlated with a high Gleason score and low CD8^+^ T-cell abundance in bone metastatic PCa 13,14, and a population of immunosuppressive myeloid-derived suppressor cells (MDSCs) (CD11b^+^Gr1^+^) massively infiltrate the mCRPC microenvironment, especially in *PTEN*-null PCa 15-17. Therefore, restoring PTEN function could not only overcome Enz resistance and bone metastasis but also reverse the immunosuppressive tumor microenvironment (TME) to achieve significant inhibition of tumor growth and metastasis 18,19.

Furthermore, immunosuppressive cytokines associated with MDSCs promote the progression of CRPC. IL-23 secreted by highly infiltrated MDSCs from tumors drives insensitivity to ADT in patients with CRPC, and IL-23 blockers or CXCR2 inhibitors can increase the Enz effect in *Pten*-deficient mice 20. Another study revealed that tumor-derived chemokines such as IL-8 and CXCL5 drive the recruitment of MDSCs into patients with Enz-treated CRPC by binding to CXCR2, highlighting the blockade of CXCR2 as a potential strategy to increase Enz sensitivity 21,22. Additionally, CXCR2 ligands have been demonstrated to be upregulated in response to the loss of tumor suppressors and oncogenic activation 15,22,23. After ADT and Enz treatment, the number of CXCR2^+^ cells in PCa increased, and targeting CXCR2 could overcome mCRPC resistance to AR inhibitors such as Enz 24,25. Our previous studies revealed that CXCR2 siRNA has much greater cytotoxicity than a small-molecule CXCR2 inhibitor (AZD5069) in PCa cells (Figure S1 and Table S1). Therefore, in this study, CXCR2 siRNA (siCXCR2) was chosen for combination with the PTEN plasmid (pPTEN) for drug-resistant and metastatic CRPC treatment.

**Scheme 1:**
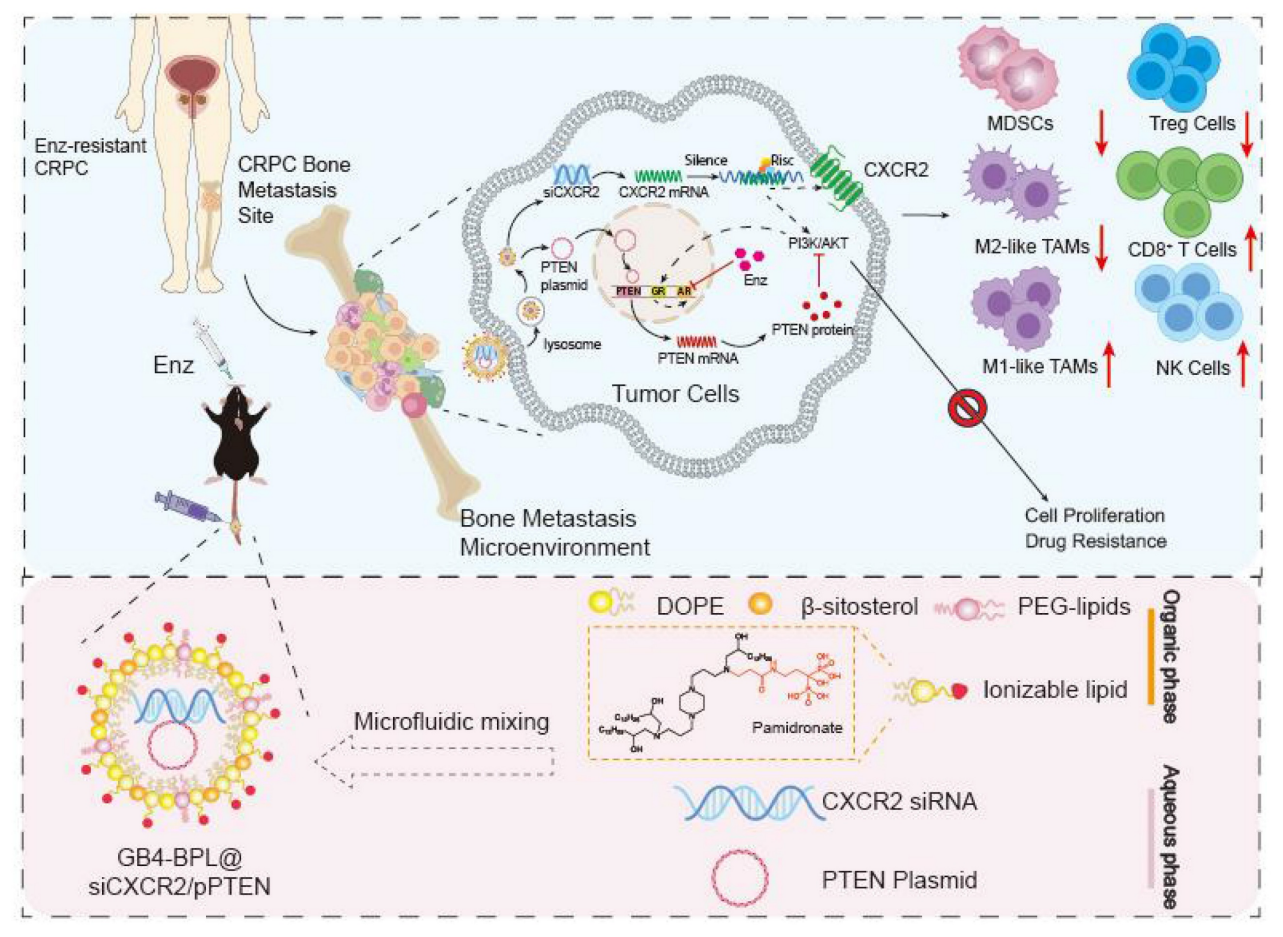
Illustration of designing GB4-BPL for targeted delivery of pPTEN and siCXCR2 to EnzR BmCRPC, which could overcome drug resistance, restrain tumor progress and metastasis as well as remold tumor immune-microenvironment.

Because nearly 90% of patients with mCRPC experience metastasis to the bone, a vector for bone-targeted delivery of siCXCR2 and pPTEN with high transfection efficiency and safety is favorable. In this study, we synthesized a pamidronate-modified ionizable lipid for bone targeting, which was further mixed with β-sitosterol, phospholipids, and PEG-lipids to form lipid nanoparticles (GB4-BPLs). Pamidronate is a type of bisphosphonate (BP), an analog of inorganic pyrophosphate, that can chelate strongly with the calcium ion (Ca^2+^) of hydroxyapatite (HA), the main inorganic component of bone, endowing pamidronate with strong affinity and rapid adsorption to the bone surface 26. We hypothesized that GB4-BPL could actively target the codelivery of pPTEN and siCXCR2 to bone metastasis sites with high efficiency and low toxicity, restoring *Pten* function and reversing the immunosuppressive tumor microenvironment. Additionally, this nanosystem has tumor-targeting ability due to the enhanced permeability and retention (EPR) effect. Therefore, siCXCR2 and pPTEN loaded in GB4-BPL (GB4-BPL@siCXCR2/pPTEN) might provide potential therapeutic options for drug-resistant BmCRPC (Scheme 1).

## Methods

### Reagents

Commercial suppliers provided all reagents and solvents, which were utilized without additional purification. N-acryloxysuccinimide (NAS, 97%) and pamidronate (98%) were purchased from Yuanye Bio-Technology Co., Ltd. (Shanghai, China). 2-(4-Methyl-piperazin-1-yl)-ethylamine (98%), 1-(3-aminopropyl) piperazine (98%), 1,4-bis (3-aminopropyl) piperazine (98%), 1,2-dioleoyl-sn-glycero-3-phosphoethanolamine (DOPE, 98%), and β-sitosterol were obtained from Aladdin. 1,2-Epoxy-dodecane (C12, 90%) and 1,2-epoxytetradecane (C14, 85%) were obtained from Sigma‒Aldrich. 1,2-Dimyristoyl-sn-glycero-3-phosphoethanolamine-N-(methoxypolyethylene glycol-2000) (C14-PEG2000) was obtained from Avanti Polar Lipids. Enzalutamide (Enz) was generously provided by Shanghai Fosun Pharmaceutical Group Co., Ltd. PTEN plasmid, EGFP plasmid, Cy3-siRNA, siFAM and CXCR2 siRNA were purchased from GenePharma (Shanghai, China). The 4% paraformaldehyde fixative solution (Servicebio), TUNEL apoptosis assay kit, Ki67 cell proliferation assay kit and Alexa Fluor 488-labeled goat anti-rabbit IgG (H + L) secondary antibody were obtained from Servicebio. A Cell Counting Kit-8 (CCK-8), propidium iodide (PI), 4',6-diamidino-2-phenylindole (DAPI), 3,3'-dioctadecyloxacarbocyanine perchlorate (DiO, λex/λem = 484/501 nm), 1,1'-dioctadecyl-3,3,3',3'-tetramethylindodicarbocyanine, 4-chlorobenzenesulfonate salt (DiD, λex/λem = 644/655 nm), LysoTracker Red (λex/λem = 577/590 nm) and an Annexin V-FITC apoptosis detection kit were purchased from Beyotime Biotechnology. 1,1-Dioctadecyl-3,3,3',3'-tetramethylindotricarbocyanine iodide (DiR, λex/λem = 750/780 nm) was obtained from Shanghai Bioscience Technology Co., Ltd.

### Cell culture

HEK-293T, RM-1, 22RV1, MC3T3-E1 and NIH3T3 cells were obtained from the Cell Bank of the Chinese Academy of Sciences (CAS, Shanghai, China). PTEN.CaP8 cells were purchased from American Type Culture Collection (ATCC, USA). RM-1 cells and PTEN.CaP8 cells were chronically exposed to RPMI-1640 medium containing Enz (20 μM) for 6 months. RM-1 cells and PTEN.CaP8 cells resistant to Enz are referred to as RM-1 EnzR and PTEN.CaP8 EnzR cells. HEK-293T cells were cultured in Dulbecco's modified Eagle's medium (DMEM) (Gibco, USA). RM-1 cells, PTEN.CaP8 cells and 22RV1 cells were cultured in RPMI-1640 medium (Gibco, USA). MC3T3-E1 cells were cultured in α-MEM (Gibco, USA). All the media were supplemented with 10% fetal bovine serum (FBS) (Gibco, USA) and 100 U/mL penicillin/streptomycin (P/S) (Gibco, USA). PTEN.CaP8 medium was supplemented with 25 µg/mL bovine pituitary extract (BPE, Absin, abs9119-20 mg), 5 µg/mL human recombinant insulin (Procell, PB180432) and 6 ng/mL human recombinant epidermal growth factor (EGF, PeproTech, AF-100-15-100 μg). NIH3T3 cells were cultured in DMEM supplemented with 10% calf serum (CS) (Gibco, USA) and 100 U/mL P/S. A cell culture incubator (Thermo) was used to perform cell culture at 37 °C under 5% CO_2_.

### Synthesis and identification of cationic lipid GB4-C14

The cationic lipid GB4-C14 was synthesized by a previously reported method 26 (Figure S2). In detail, first, N-acryloxysuccinimide (NAS) (101.48 mg, 0.6 mmoL, 1.2 equiv.), pamidronate sodium (135 mg, 0.5 mmoL, 1.0 equiv.), and diisopropylethylamine (DIPEA, 129.24 mg, 1 mmoL, 2.0 equiv.) were mixed in a flame-dried 50 mL round-bottom flask with 2 mL of deionized water and ethanol. After reacting overnight at 40 °C, the solvent was removed by vacuum distillation to obtain the crude product, which was then rinsed with dichloromethane followed by filtration through silica gel. Then, water was added to the solid until it was completely dissolved, and acetone was slowly added to precipitate the crystals. Second, pamidronate acrylamine (100 mg, 0.307 mmoL, 1 equiv.) and 1,4-bis(3-aminopropyl) piperazine (224.8 mg, 1.538 mmoL, 5 equiv.) were dissolved in deionized water (300 μL), mixed in water and stirred overnight at room temperature. Then, water was added to the solvent until it was completely dissolved, and acetone was slowly added to precipitate the crystals twice. Then, Compound 2 was obtained after centrifugation and drying. Finally, Compounds 2 (9.85 mg, 0.015 mmoL, 1 equiv.) and C14 (15.29 mg, 0.072 mmoL, 4.8 equiv.) were dissolved in ethanol/water (1/1), mixed and stirred at 80 °C for 3 days before being cooled to ambient temperature. Then, water was added to the solvent until it was completely dissolved, and acetone was slowly added to precipitate the crystals. The final product was obtained by recrystallization. The compounds were characterized using hydrogen nuclear magnetic resonance (NMR) and tandem mass spectrometry (MS).

### Preparation and characterization of GB4-BPL for nucleic acid delivery

The GB4-BPL was prepared using a microfluidic method in a microfluidic device (NEXSTAR NANO3, Shanghai, China). In brief, a DNA plasmid (pPTEN or pEGFP) and siRNA in 10 mM citrate buffer solution were added to a mixture of lipids in ethanol. The mixture of lipids contained pamidronate-bearing ionizable lipids or normal ionizable lipids, DOPE, β-sitosterol and C14-PEG2000 with fixed molar ratios of 35%, 16%, 46.5% and 2.5%, respectively. The ethanol and aqueous solution were mixed at flow rates of 1 and 3 mL/min (1:3) using a NEXSTAR C2 chip. Then, the samples were concentrated, followed by ultrafiltration using a Milipore 30 KD ultrafiltration tube (30,000 MWCO, Merck). The GB4-BPL were subsequently stored in PBS containing 10% sucrose at -80 °C for further use.

The morphology of GB4-BPL was determined using transmission electron microscopy (TEM, 120 KV, Talos, FEI, USA) after negative staining with saturated uranyl acetate solution. Moreover, the nanoparticle size distribution and zeta potential values were analyzed using a Nano ZS90 instrument (Malvern, England). Additionally, the GB4-BPL were stored in PBS (pH 7.4) or PBS (pH 7.4) containing 10% FBS at 4 °C for 72 h to evaluate their stability in simulated physiological fluids.

### Gene transfection assays

To evaluate the transfection efficiency of GB4-BPL, HEK-293T and RM-1 EnzR cells were seeded in 24-well plates. Subsequently, pEGFP was co-incubated with GB4-BPL (GB4-BPL@pEGFP) at different mass ratios, and Lipo8000@pEGFP was used as a control. After co-culturing with cells, the fluorescence intensity of each group was observed in HEK-293T using fluorescence microscope (OLYMPUS, CKX53), and in HEK-293T cells and RM-1 EnzR cells using a fluorescence microplate reader (Biotek Synergy H1).

### *In vitro* cellular uptake assay and intracellular colocalization

To assess the cellular uptake efficiency, GB4-BPL@siFAM (20 nM) and GB4-BPL@Cy3-siRNA (20 nM) were prepared. RM-1 EnzR and PTEN.CaP8 EnzR cells were seeded in 24-well plates and incubated overnight. Then, cells were incubated with each group, and the cellular uptake in each group was evaluated via CytoFlex S flow cytometry (Beckman, USA) for 1 and 4 h and subsequently analyzed in FlowJo software. For fluorescence imaging, RM-1 EnzR cells were incubated with DiO-labeled GB4-BPL@Cy3-siRNA for 1, 4 and 6 h, and cellular uptake was visualized using fluorescence microscope.

After 4 h of coincubation with DiO-labeled GB4-BPL@Cy3-siRNA, the cells were fixed with paraformaldehyde, and then, a sealing solution containing DAPI was applied to the coverslip. The intracellular distribution was observed using a spinning disk confocal superresolution microscope (SpinSR10, Olympus).

### Lysosome escape assay

To investigate the lysosomal escape capability, GB4-BPL@siFAM (20 nM) was prepared. RM-1 EnzR cells were seeded in 24-well plates. After 24 h of culture, the RM-1 EnzR cells were coincubated with GB4-BPL@siFAM for 1 h and 4 h and then labeled with LysoTracker Red (50 nM) to label the lysosomes. DAPI was used for cell nucleus staining. Images were acquired using a spinning disk confocal superresolution microscope (SpinSR10, Olympus) after 1 and 4 h of incubation.

### *In vitro* cellular toxicity assessment of GB4-BPL

NIH3T3, RM-1 EnzR, and PTEN.CaP8 EnzR cells were seeded in 96-well plates at a density of 5000 cells/well and cultured overnight with 100 μL of medium containing 10% FBS. A CCK-8 kit was used to evaluate the cytotoxicity of each group, as measured at 450 nm (OD) by a microplate reader (MULTISKAN MK3, Thermo, USA). The concentration gradient of blank GB4-BPL was 0-3 mg/mL. The following formula was used to calculate cell viability: cell viability (%) = (OD_treatment_ - OD_blank_)/(OD_control_ - OD_blank_) × 100%.

### Cellular antiproliferation and apoptosis assays

RM-1 EnzR, PTEN.CaP8 EnzR and 22RV1 cells were seeded in 96-well plates at a density of 5000 cells/well and cultured overnight. Then, the cells were added to various concentrations of the formulations and cocultured for 48 h. A CCK-8 kit was used to evaluate the cytotoxicity in each group, as measured at 450 nm (OD) with a microplate reader. The concentrations of pPTEN/siCXCR2 were 0-3 μg/mL and 0-40 nM, respectively. The Enz concentrations ranged from 0-100 μg/mL. For the cell apoptosis assay, RM-1 EnzR cells were seeded in 6-well plates at a density of 3 × 10^5^ cells/mL and cultured overnight. RM-1 EnzR cell apoptosis rates were detected using an Annexin V-FITC/PI double-staining assay kit by flow cytometry (siCXCR2: 8.3 nM, pPTEN: 1.156 μg/mL, Enz: 30 μg/mL).

### Cell anti-migration and anti-invasion assays

RM-1 EnzR cells were cultivated with serum-free medium for 24 h. Then, RM-1 EnzR cells were seeded in the upper chamber of a Transwell plate (8-μm, Corning) at a density of 10^5^ cells/mL, and DMEM (800 μL) containing 20% FBS was added as a chemokine to the lower chamber. Matrigel (Beyotime) was added to the upper chamber of the Transwell plate. After coincubation with each group (Enz: 14.16 µg/mL; siCXCR2: 2.1755 nM; pPTEN: 0.354 μg/mL) for 24 h (anti-migration assay) or 48 h (anti-invasion assay), the cells on the surface of the upper chamber were carefully removed with a sterile cotton swab. The cells were fixed with 4% paraformaldehyde for 30 min, stained with 0.1% crystal violet (Beyotime) for 20 min and washed 3 times with PBS. The migratory/invasive cells were observed and photographed under a bright field using a fluorescence microscope (DP80, Olympus). The number of migratory/invasive cells in the images was calculated using ImageJ software.

### Western blotting

Total cell lysates were collected in RIPA buffer (Beyotime Biotech, China). The protein concentration was determined with a BCA assay kit (Beyotime Biotech, China). After dilution in loading buffer at a ratio of 4:1, the lysates were heated and denatured at 95 ℃ for 5 minutes. Then, the protein samples were loaded for SDS‒PAGE and transferred onto PVDF membranes (Immobilon^®^-P, Millipore, USA). The membranes were blocked in QuickBlock™ Blocking Buffer (Beyotime Biotech, China) and probed with primary antibodies (E-cadherin: Wanleibio, WL01482, 1:2000, N-cadherin: ABclonal, A19083, 1:1000 and Vimentin: ABclonal, A19607, 1:20000). HRP-conjugated goat anti-rabbit IgG (H+L) was used as a secondary antibody at a dilution of 1:10,000 (ABclonal, AS014). The blots were detected with an enhanced chemiluminescence (ECL) detection kit (Beyotime Biotech, China).

### Quantitative real-time PCR

TRIzol (Vazyme, China), HiScript III All-in-one RT SuperMix Perfect (Vazyme, China) and Taq Pro Universal SYBR qPCR Master Mix (Vazyme, China) were used according to the manufacturers' instructions. The qPCRs (SYBR system; Bio-Rad) for each sample were conducted in triplicate. The primer sequences were obtained from PrimerBank (http://pga.mgh.harvard.edu/primerbank/index.html). Gene expression levels were normalized to GAPDH expression and analyzed using the 2^-ΔΔCt^ method. The sequences of the primers used were as follows: PTEN forward, 5'-TGGATTCGACTTAGACTTGACCT-3'; PTEN reverse, 5'-GCGGTGTCATAATGTCTCTCAG-3'; AR forward, 5'-CTGGGAAGGGTCTACCCAC-3'; AR reverse, 5'-GGTGCTATGTTAGCGGCCTC-3'; CXCR2 forward, 5'-ATGCCCTCTATTCTGCCAGAT-3'; CXCR2 reverse, 5'-GTGCTCCGGTTGTATAAGATGAC-3'; GAPDH forward, 5'-AGGTCGGTGTGAACGGATTTG-3'; and GAPDH reverse, 5'-TGTAGACCATGTAGTTGAGGTCA-3'.

### Formation and characterization of three-dimensional (3D) multicellular tumor spheroids

The preparation of RM-1 EnzR and MC3T3 multicellular spheroids was accomplished through the liquid overlay method. In brief, sterile agarose (50 μL per well) was added to 96-well plates. Subsequently, DiO-stained RM-1 EnzR cells and DiD-stained MC3T3 cells (1 × 10^4^ cells each) were proportionally distributed in a 96-well plate and then subjected to centrifugation at 1500 rpm for 12 min. During the spheroid formation process, the culture medium (DMEM) was refreshed every 3 days. The growth of the tumor spheroids was monitored using a spinning disk confocal super-resolution microscope (SpinSR10, Olympus).

### Penetration of GB4-BPL in 3D multicellular tumor spheroids

The multicellular tumor spheroids were prepared as described above, but without the addition of dye. GB4-BPL was then stained with DiO at room temperature for 20 minutes. Using Cy7-DNA as a model drug, GB4-BPL-DiO@Cy7-DNA was prepared and co-cultured with tumor spheroids for 8 hours. After incubation, the spheroids were washed three times with PBS and fixed with 4% paraformaldehyde for 30 minutes. DAPI was used for nuclear staining. These spheroids were then transferred to a confocal dish, and images were captured by spinning disk confocal super-resolution microscopy using Z-stack imaging from the bottom of the spheroids. Image processing was performed using ImageJ software.

### *In vivo* biodistribution study

Six-week-old C57BL/6J male normal mice (18-22 g) were obtained from the Experimental Animal Center of Fudan University (Shanghai, China). All experiments were carried out in accordance with the relevant ethics principles of the Fudan University Experimental Animal Ethics Committee (2022-03-LY-GY-48). To establish the RM-1 EnzR BmCRPC mouse model, RM-1 EnzR cells were injected into the marrow cavity at 1 × 10^6^ cells per mouse. Briefly, the mice were narcotized and fixed, the femur and tibia of the right hind limb were bent at 90°, and a 1 mL sterile syringe was inserted into the bone marrow cavity along the long axis of the tibia.

A mouse model of EnzR BmCRPC was established to investigate the *in vivo* distribution of GB4-BPL, with DiR serving as a model drug. This was achieved by intravenously injecting free DiR, DiR-loaded GB4-lipo (GB4-lipo@DiR) and DiR-loaded GB4-BPL (GB4-BPL@DiR) at a concentration of 1 mg/kg. The mice were subjected to scanning at 0, 2, 4, 8, 12, and 24 h using an *in vivo* imaging system (IVIS Spectrum, Perkin Elmer), after which they were euthanized at 24 h. *Ex vivo* imaging was performed on the heart, liver, spleen, lung, kidney, tumor, left leg and right leg after resection. All the data were subsequently analyzed using Living Image^@^ 4.4 software. Additionally, cryosections were made at a thickness of 10 μm (Leica 1950, Germany), the tumor sections were counterstained with DAPI, and the fluorescence signals in the whole tumor sections were recorded with a fluorescence microscope (DM 6B, Leica, Germany). Afterward, the fluorescence signals were analyzed using ImageJ software.

### *In vivo* therapeutic efficacy in an EnzR BmCRPC mouse model

For the *in vivo* therapeutic efficacy of GB4-BPL@siCXCR2/pPTEN, a mouse model of EnzR BmCRPC was established as described above. The castration surgery was conducted 2 days after tumor implantation. For GB4-BPL@siCXCR2/pPTEN treatment, the mice were randomly divided into 8 groups (n = 6): (a) PBS; (b) blank vehicle without pamidronate (free vehicle); (c) GB4-BPL; (d) GB4-BPL@siCXCR2; (e) GB4-BPL@pPTEN; (f) GB4-BPL@siCXCR2/pPTEN; (g) Enz; and (h) Enz+GB4-BPL@siCXCR2/pPTEN (pPTEN: 0.7 mg/kg, siCXCR2: 0.5 mg/kg, Enz: 10 mg/kg). When the tumor sizes reached approximately 20-30 mm^3^, the mice were subjected to tail vein injection every 3 days for 2 weeks (Enz, oral gavage, once daily for 2 weeks). Body weights and tumor sizes were measured every 2 days, and average tumor volumes were calculated according to the following formula: V = L × W^2^/2, where 'L' represents the length of the longest axis of the tumor and 'W' represents the length of the axis perpendicular to the longest axis. Fourteen days following the first injection, all the animals were euthanized, and the tumor tissues were harvested and weighed. Both tumors and organs were collected for subsequent histological examination.

### Survival study

Once the tumor volume reached 20-30 mm^3^, the mice were randomly divided into five groups (n = 4) and treated by tail vein injection every 3 days for 2 weeks with different formulations. The body weights and tumor sizes of the mice were measured every 2 days for survival studies. In accordance with animal ethics standards, the mice were euthanized when the tumor volumes reached 2000 mm^3^.

### Immunofluorescence, H&E, TRAP, and OPG staining and BMP-2 staining for tissue and blood sample analysis

The mice were euthanized at the end of treatment. The tumors, tumor bearing tibias and various organs (lung, heart, liver, kidney, and spleen) were harvested and fixed with 4% paraformaldehyde, embedded in paraffin and sectioned into slices at a thickness of 5 μm. Hematoxylin and eosin (H&E) staining was performed on the tissue sections for microscopic examination. Trap staining, OPG staining and BMP-2 staining of tumor-bearing tibias were performed to detect bone injury. For immunofluorescence staining, tumor samples were incubated with different primary rabbit antibodies (PTEN, CD4, CD8, CD11b, and Gr-1) at a 1:50 dilution overnight at 4 °C, washed with PBS, and incubated with fluorescently labeled secondary antibodies (1:1000) for 60 min at room temperature. Finally, the slides were imaged using a confocal microscope (Olympus FluoView FV1000). Additionally, blood samples were collected from the retro-orbital venous plexus to assess biochemical indicators (ALT, AST, BUN, and CREA).

### Immunomodulatory efficacy analysis

The immunological efficacy of GB4-BPL@siCXCR2/pPTEN in relieving tumor immunosuppression and boosting antitumor immunity was investigated in BmCRPC. Tumor tissues were collected to examine the immunomodulatory effects. The tumor tissues from each group were cut into small pieces and incubated in digestion medium consisting of 0.2 mg/mL collagenase IV, 0.1 mg/mL DNase I and 0.2 mg/mL hyaluronidase for 2 h at 37 °C to obtain a single-cell suspension. To evaluate the effects on immune cells such as MDSCs, Tregs, macrophages, and NK and CD8^+^ T lymphocytes in tumors, the isolated cells from each group were stained as described below and then incubated with typical antibodies for flow cytometry analysis (n = 4). For MDSCs, the isolated cells were stained with 7-aminoactinomycin D (7-AAD) and incubated with anti-CD11b PerCP-Cy5.5 and anti-Ly-6G/Ly-6C(Gr-1)-PE, and the resulting MDSCs were denoted as CD11b^+^Gr-1^+^ cells. For Tregs, the isolated cells were stained with fixable viability dye (FVD) eFluor 450 and anti-CD45-Alexa Fluor 700 and then incubated with anti-CD3ε-FITC, anti-CD4-APC, and anti-Foxp3-PerCP-Cy5.5. The proportion of Tregs was determined as CD45^+^CD3^+^CD4^+^Foxp3^+^ cells. For NK cells, the isolated cells were stained with 7-AAD and incubated with anti-CD3ε-FITC and anti-CD49b-PE, and the resulting NK cells were denoted as CD3^-^CD49b^+^ cells. For CD8^+^ T lymphocytes, the isolated cells were incubated with FVD eFluor 450, anti-CD45-Alexa Fluor 700, anti-CD3ε-FITC and anti-CD8a-PE to identify the CD8^+^ T lymphocytes. The effects on the phenotypes of macrophages in tumors were investigated, and the isolated cells from each group were stained with anti-F4/80-FITC, anti-CD11b-PerCP-Cy5.5, anti-CD206-PE and anti-CD86-PE-Cy7 antibodies. The proportions of M2 macrophages (CD11b^+^F4/80^+^CD86^-^CD206^+^) and M1 macrophages (CD11b^+^F4/80^+^CD86^+^CD206^-^) were determined by flow cytometry.

### *In vivo* effects on inhibiting cell proliferation and inducing apoptosis

For TUNEL assay detection, the tumor tissues from each group were paraffin-embedded and sectioned at 5 μm for TUNEL assay detection. Following proteinase K repair, the sections were permeabilized for 10 min at RT. Next, the reaction mixture was added, and the sections were incubated at 37 °C for 2 h. Finally, the cell nuclei were stained with DAPI by incubation in the dark at RT for 10 min. Tumor tissue sections were stained with an anti-Ki67 primary antibody (1:200 dilution), followed by an Alexa Fluor 488-labeled goat anti-rabbit IgG (H+L) secondary antibody (1:400 dilution) to assess the proliferation of tumor cells. The cell nuclei were stained with DAPI. The sections were then observed under a fluorescence microscope to capture images. DAPI emits blue light with an excitation wavelength of 330-380 nm and an emission wavelength of 420 nm; Alexa Fluor 488 emits green light with an excitation wavelength of 488 nm and an emission wavelength of 519 nm. ImageJ software was used to analyze the TUNEL and Ki67 signals in the captured images.

### Micro-CT imaging and analysis of bone loss

The right hind limb tibias were collected, and normal left hind limbs were used as controls (n = 4). Bone images were obtained using microCT (Siemens Inveon microCT, SD_000_N8-875, Germany) under 80 kVp conditions. A tissue-equivalent phantom (Siemens, Germany) was used to establish a standard line of bone mineral density (BMD), which was used to convert the heat unit (HU) values detected by microCT to BMD values (mg·cc^-1^). Four areas of each tibia were randomly selected to measure the HU values and adjusted to the BMD values according to the standard line.

### Statistical analysis

Statistical analysis was performed using GraphPad Prism^®^ 8 software. Two-tailed Student's *t* test was applied when there were only two groups of samples. The results are presented as the means ± SDs. Comparisons were performed using Student's *t* test for comparisons between two groups or one-way analysis of variance (ANOVA) for comparisons among multiple groups. Statistical differences were significant at *p < 0.05 and very significant at **p < 0.01, ***p < 0.001, ****p < 0.0001.

## Results

### Synthesis and characterization of GB4-BPL

**Figure 1:**
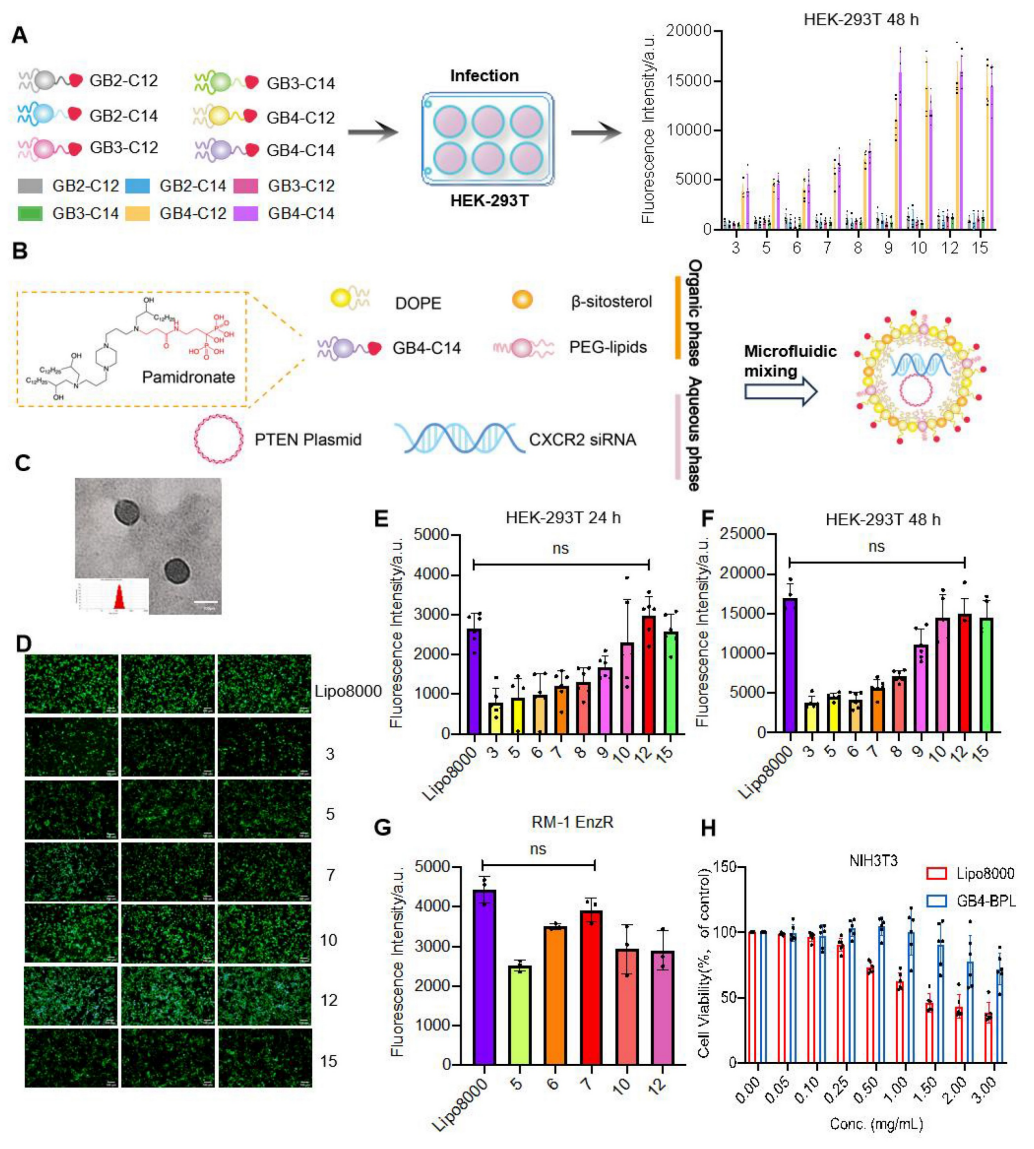
Synthesis and characterization of GB4-BPL.** (A)** Schematic diagram of BP-lipid nanoparticles screening for efficient gene delivery to HEK-293T cells. Fluorescence intensity expression in HEK-293T cells after treatment with the BP lipids encapsulating EGFP plasmid. 5000 cells were incubated with lipid nanoparticles at a EGFP plasmid dose of 10 ng/well (n = 3, mean ± SD). **(B)** Schematic illustration of synthesizing GB4-BPL. **(C)** TEM images of GB4-BPL; scale bars = 100 nm. **(D-F)** Transfection assessments of GB4-BPL@pEGFP complexes on HEK-293T cells at different mass ratios. **D)** Images of fluorescence microscope; scale bars = 50 μm. **E, F)** Fluorescence intensity results of fluorescence microplate reader at 24, 48 h (n = 4, mean ± SD), ns: no significance, one-way ANOVA. **(G)** Transfection assessments of GB4-BPL@pEGFP complexes on RM-1 EnzR cells at 48 h, Lipo8000@pEGFP as control (n = 3, mean ± SD), ns: no significance, one-way ANOVA.** (H)** Cytotoxicity of GB4-BPL at different concentrations in NIH3T3 cells, Lipo8000 was used as control, GB4-BPL: 0~3 mg/mL (n = 6, mean ± SD, one-way ANOVA).

A series of bisphosphonate (BP) lipid materials consisting of pamidronate were synthesized, with specific synthesis steps and materials provided in Figure S2. Initially, pamidronate sodium was reacted with N-acryloxysuccinimide (NAS) to synthesize pamidronate acrylamide, followed by conjugation onto amine cores through a Michael addition reaction to obtain pamidronate-bearing amine cores. Subsequently, six BP lipids were obtained by reacting these amidronate-bearing amine cores with epoxy-terminated alkyl chains. Furthermore, these BP lipids were mixed with phospholipid DOPE, β-sitosterol, lipid-anchored poly (ethylene glycol) (C14-PEG2000), and an EGFP plasmid using a microfluidic device to prepare 6 lipid nanoparticles. Among these, the lipid GB4-C14 exhibited the optimal transfection efficiency in gene transfection experiments (Figure 1A). The characterization of GB4-C14 is provided in the Supplementary material (Figures S3-S8). Furthermore, GB4-BPL was fabricated with GB4-C14, DOPE, β-sitosterol, and C14-PEG2000 using a microfluidic device (Figure 1B). A counterpart liposomal formulation without pamidronate (GB4-lipo) and commercially available liposomes (Lipo8000) were developed as controls. The TEM results indicated that the GB4-BPL could form nanometer-sized spherical particles (Figure 1C). The dynamic light scattering (DLS) analysis indicated that GB4-BPL exhibited a Z-average diameter of nearly 145.1 nm with a polydispersity index (PDI) of 0.186. In addition, GB4-BPL exhibited neutral surface charges with ζ potential values between -2 mV and +2 mV. When GB4-BPL was incubated in mimicked physiological fluids consisting of PBS (pH 7.4) containing 10% fetal bovine serum (FBS) for 72 h, the average diameter and ζ potential values of GB4-BPL rarely changed (Figure S9). These data verified the good stability of GB4-BPL in the mimicked physiological fluids.

Moreover, the resistance index of Enz-resistant cells was evaluated. For this purpose, RM-1 and PTEN.CaP8, and RM-1 EnzR (PTEN-deficient RM-1 cells with Enz resistance), PTEN.CaP8 EnzR cells were coincubated with different concentrations of Enz for 48 h, and the IC_50_ values were determined using a CCK-8 kit. The results revealed that the IC_50_ of RM-1 EnzR cells was 6.625 times greater than that of RM-1 cells, and the IC_50_ of PTEN.CaP8 EnzR cells was 5.151 times greater than that of PTEN.CaP8 cells (Figure S10 and Table S2). These findings indicated that both drug-resistant cell lines were ready for further use, as their resistance indices (RIs) exceeded 5. Moreover, the quantitative real-time PCR results revealed the mRNA expression levels of AR in RM-1 EnzR and PTEN.CaP8 EnzR cells were much higher than those in RM-1 and PTEN.CaP8 cells (Figure S11), which demonstrated that RM-1 EnzR and PTEN.CaP8 EnzR cells were resistant to Enz, and *Pten* was deficient in RM-1 EnzR cells 5.

Next, the gene transfection efficiencies of GB4-BPL in HEK-293T cells and RM-1 EnzR cells were evaluated by fluorescence microscopy and in a microplate reader with pEGFP as a model drug. The results revealed that a notably high transfection efficiency was achieved at a mass ratio of 12 in HEK-293T cells at 24 and 48 h, which was equivalent to that of Lipo8000 (*p* > 0.05) (Figure 1, D-F). Moreover, the highest transfection efficiency was observed at a mass ratio of 7 in murine PCa RM-1 EnzR cells at 48 h (Figure 1G). These results indicated that GB4-BPL could be used for gene delivery with a transfection efficiency as high as that of Lipo8000.

Furthermore, the cytotoxicity of GB4-BPL was evaluated in NIH3T3 murine fibroblasts in a CCK-8 assay, with Lipo8000 used as a control. The results revealed no obvious cytotoxicity when the concentration of GB4-BPL was increased to 1.5 mg/mL (*p* > 0.05). However, there was significant cytotoxicity when the concentration of Lipo8000 was increased to 0.5 mg/mL, with only 75% cell viability (Figure 1H) (*p* < 0.05), which demonstrated the much greater biocompatibility of GB4-BPL compared with that of Lipo8000. Similar results were shown for RM-1 EnzR and PTEN.CaP8 EnzR cells (Figure S12).

### GB4-BPL enhanced tumor spheroid penetration and promoted lysosomal escape in EnzR cells with high transfection ability

**Figure 2:**
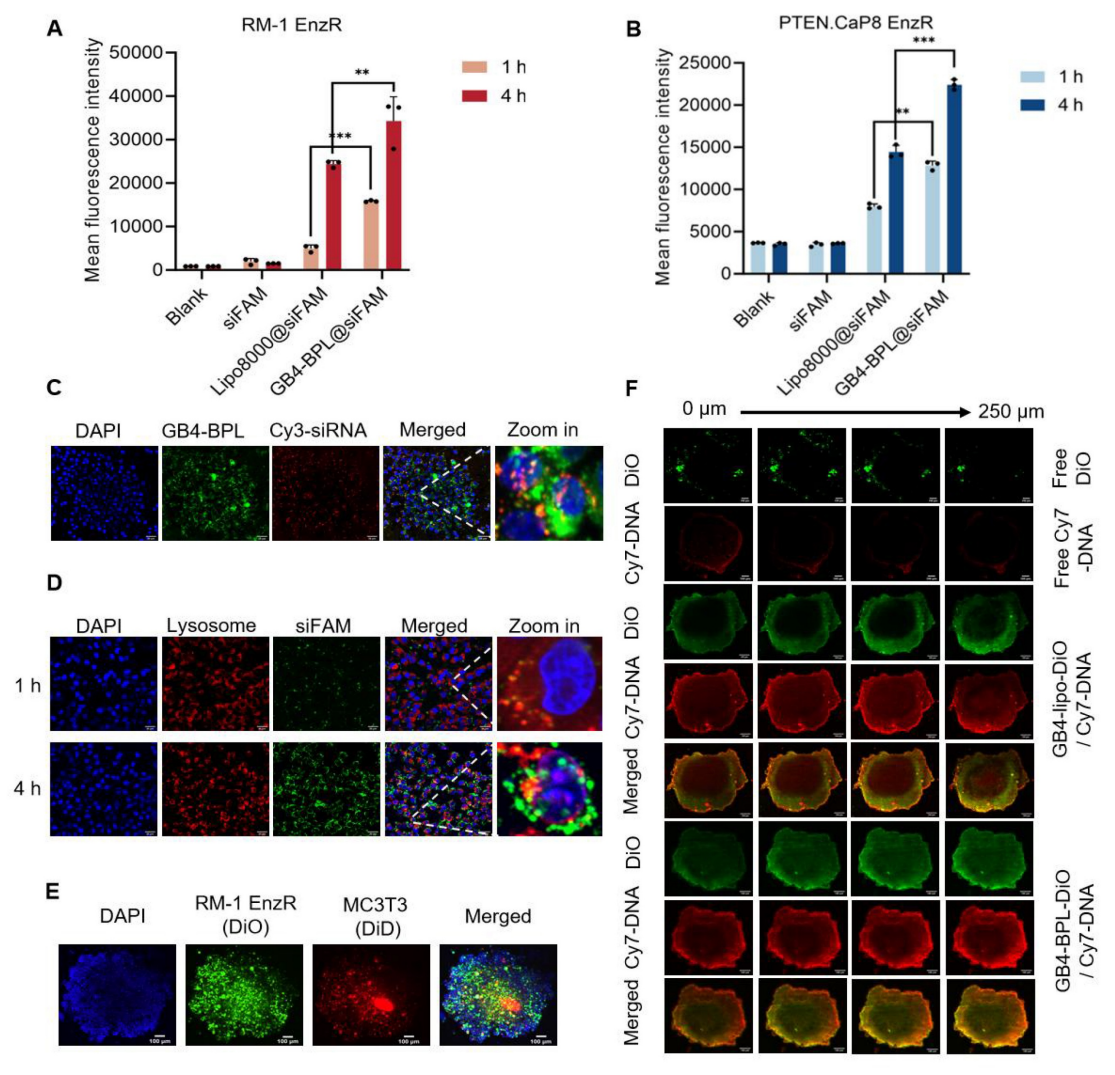
The cellular uptake of GB4-BPL *in vitro*. **(A, B)** Cellular uptake of GB4-BPL detected by flow cytometry in RM-1 EnzR cells and PTEN.CaP8 EnzR cells at 1 h and 4 h, siFAM: 20 nM. (n = 3, mean ± SD), *p < 0.05, **p < 0.01, ***p < 0.001, one-way ANOVA. **(C)** Images of intracellular distribution. Cy3-siRNA: 20 nM, scale bars = 20 μm. **(D)** Images of lysosome escape. siFAM: 20 nM, Lysotracker red: 50 nM, scale bars = 20 μm. **(E)** 3D tumor spheroid model. DiO-stained RM-1 EnzR cells and DiD-stained MC3T3 cells were seeded in the 96-well plates at the ratio of 1:1. Blue fluorescence: DAPI, green fluorescence: DiO, red fluorescence: DiD, scale bars = 100 μm. **(F)** The images of *in vitro* tumor spheroids penetration of each group were acquired at 10× objective magnification using Z-stack imaging, scale bars = 100 μm.

Next, the cellular uptake of GB4-BPL into RM-1 EnzR cells was evaluated using flow cytometry and fluorescence microscopy, with Cy3-siRNA and FAM-siRNA used as the model drugs. The results revealed that the Cy3-siRNA signal appeared in the cytoplasm at 1 h and began to appear in the nucleus at 4 h. Moreover, the Cy3-siRNA signal was restored from the nucleus to the cytoplasm at 6 h (Figure S13). After 1 and 4 h, the mean fluorescence intensities of GB4-BPL@siFAM in the RM-1 EnzR cells were 1.68- and 1.65-fold greater than those of Lipo8000@siFAM, respectively (Figure 2A). Similarly, in PTEN.CaP8 EnzR cells, the mean fluorescence intensities of GB4-BPL@siFAM were 1.66- and 1.59-fold greater than those of Lipo8000, respectively (Figure 2B). These results showed that GB4-BPL could enhance the cellular uptake of gene drugs. Moreover, the intracellular colocalization of GB4-BPL@Cy3-siRNA was investigated by spinning disk confocal superresolution microscopy. As shown in Figure 2C, the red fluorescence (Cy3-siRNA) and green fluorescence (GB4-BPL) were distributed mainly around the nuclei (DAPI blue staining) and merged into orange fluorescence in the cytoplasm. Notably, excellent lysosomal escape is vital to the bioavailability and pharmacodynamics of nucleic acids, as genetic drugs are highly susceptible to phagocytic degradation by the intracellular lysosomal system 27,28. Confocal microscopy revealed that the green fluorescence of siFAM overlapped with the red fluorescence of the lysosomes around the blue fluorescence of the nucleus after 1 h of incubation. After 4 h, separation of green and red fluorescence was observed, indicating the high lysosomal escape capacity of the GB4-BPL (Figure 2D).

These findings demonstrated that GB4-BPL could help cross the lysosomal barrier and deliver encapsulated agents into the cytoplasm, thereby increasing the efficiency of targeted drug delivery and preventing the deactivation and degradation of DNA and siRNA before arriving at effect sites.

Furthermore, a 3D tumor spheroid model containing RM-1 EnzR cells and MC3T3-E1 mouse embryonic osteoblast cells was established to mimic the BmCRPC tumor environment. As shown in Figure 2E, free DiO and free Cy7-DNA were predominantly localized around the spheroids. In the GB4-BPL-DiO@Cy7-DNA group, DiO and Cy7-DNA penetrated the spheroids at a depth of 250 μm, and the fluorescence intensity was markedly greater than that in the free group (Figure 2F, S14), indicating the superior tumor penetration ability of GB4-BPL.

### GB4-BPL@siCXCR2/pPTEN inhibited cell growth and migration and enhanced the effect of Enz *in vitro*

**Figure 3:**
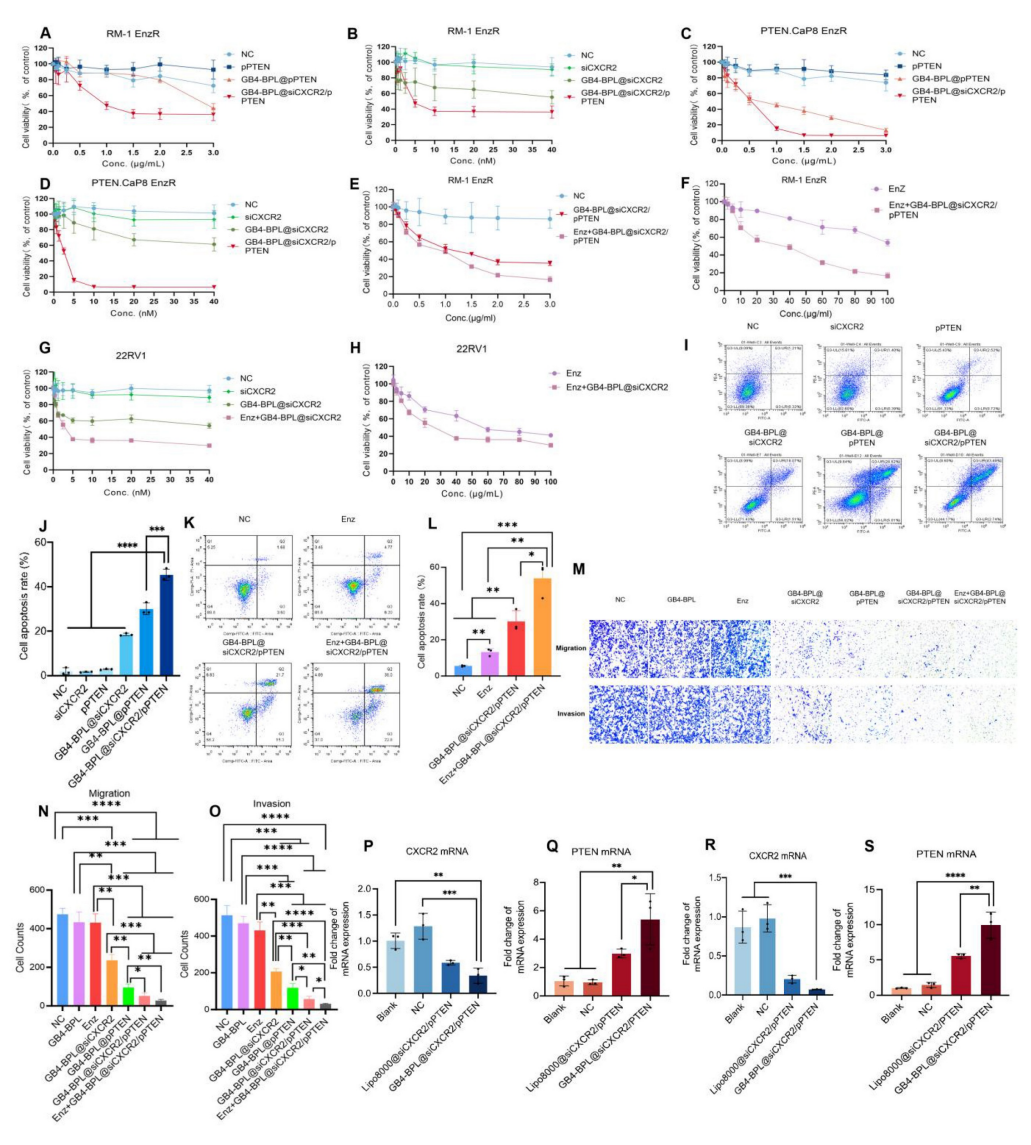
*In vitro* anti-proliferation ability tests. **(A-F)** The cytotoxicity results of each group in **A, B)** RM-1 EnzR cells for 48 h (n = 6), **C, D)** PTEN.CaP8 EnzR cells for 48 h (n = 6) and **E, F)** RM-1 EnzR cells for 48 h (n = 4). **(G, H)** 22RV1 cells for 48 h (n = 4) (siCXCR2: 0~40 nM, pPTEN: 0~3 μg/mL, Enz: 0~100 μg/mL).** (I)** The representative results of apoptosis of RM-1 EnzR cells treated with each group for 48 h (siCXCR2: 8.3 nM, pPTEN: 1.156 μg/mL). **(J)** Statistical results of cell apoptosis in each group (n = 3). **(K)** The representative results of apoptosis of RM-1 EnzR cells treated with each group for 48 h (Enz: 30 µg/mL, siCXCR2: 8.3 nM, pPTEN: 1.156 μg/mL). **(L)** Statistical results of cell apoptosis in each group (n = 3). **(M)** RM-1 EnzR cells were incubated with each group for 24 h (anti-migration assay) and 48 h (anti-invasion assay, with matrix gel to mimic tumor extracellular matrix), Enz: 14.16 μg/mL, siCXCR2: 2.1755 nM, pPTEN: 0.354 μg/mL. (scale bars = 50 μm). **(N, O)** Statistical results of anti-migration assay and anti-invasion assay in each group in each group (n = 3). **(P-S)** The RT-qPCR results of the expression of CXCR2 and PTEN mRNA in **P, Q)** RM-1 EnzR cells and **R, S)** PTEN.CaP8 EnzR cells (n = 3). Data are represented as means ± SD. *p < 0.05, **p < 0.01, ***p < 0.001, ****p < 0.0001, ns: no significance, one-way ANOVA.

To evaluate the* in vitro* cytotoxicity of GB4-BPL@siCXCR2/pPTEN, RM-1 EnzR cells, PTEN.CaP8 EnzR cells, and 22RV1 cells were treated with different formulations. As shown in Figure 3, A-D, GB4-BPL@siCXCR2, GB4-BPL@pPTEN and GB4-BPL@siCXCR2/pPTEN had significant cytotoxic effects on both cell lines at increasing concentrations. GB4-BPL@siCXCR2/pPTEN had the greatest effect in RM-1 EnzR and PTEN.Cap8 EnzR cells, which had 32.5- and 1.5-fold lower IC_50_ values than those of the single treatment, respectively. In particular, GB4-BPL@siCXCR2/pPTEN (siCXCR2/pPTEN: IC_50_ = 2.224 nM/0.4321 μg·mL^-1^) exhibited the greatest cytotoxicity toward PTEN.CaP8 EnzR cells (Table S3) among all the tested cell models. Moreover, the combination index (CI) calculated using CompuSyn software (CI < 1) indicated that the combination of siCXCR2 and pPTEN had synergistic effects on RM-1 EnzR cells and PTEN.CaP8 EnzR cells (Figure S15).

The combined effects of Enz and GB4-BPL@siCXCR2/pPTEN on the RM-1 EnzR cell model were then evaluated. As shown in Figure 3, E and F, the Enz+GB4-BPL@siCXCR2/pPTEN group exhibited much greater cytotoxicity than the GB4-BPL@siCXCR2/pPTEN (*p* < 0.01) and Enz alone (*p* < 0.001) groups. GB4-BPL@siCXCR2/pPTEN significantly enhanced the cytotoxicity of Enz against RM-1 EnzR cells, with the Enz IC_50_ decreasing from 140.3 µg/mL to 28.32 µg/mL (Table S4). The CI values demonstrated that the combination of Enz with GB4-BPL@siCXCR2/pPTEN had a synergistic effect on RM-1 EnzR cells (CI < 1) (Figure S16). These results suggest that the combination of siCXCR2 and pPTEN could enhance the effect of Enz and reduce its drug resistance in Enz-resistant CRPC. Furthermore, the cytotoxicity of GB4-BPL@siCXCR2 in a 22RV1 cell model (pPTEN-intact) with and without Enz was evaluated (Table S3 and Figure 3, G and H). The results showed that GB4-BPL@siCXCR2 also inhibited cell proliferation (IC_50_ = 64.53 nM) and had a significant synergistic effect with Enz (4.657 nM).

In addition, the average apoptosis rates of RM-1 EnzR cells after different formulation treatments were measured by flow cytometry. As shown in Figure 3, I and J and Figure S17, the average apoptosis rate of RM-1 EnzR cells treated with GB4-BPL@siCXCR2/pPTEN was the highest and reached 45.44%, which was 1-3 times greater than those of GB4-BPL@siCXCR2 and GB4-BPL@pPTEN alone. Enz+GB4-BPL@siCXCR2/pPTEN increased the apoptosis rate in RM-1 EnzR cells as high as 53.8%, which was 4.07- and 1.78-fold greater than those of the Enz and GB4-BPL@siCXCR2/pPTEN groups, respectively (Figure 3, K and L and Figure S18). These results indicated that GB4-BPL@siCXCR2/pPTEN significantly increased the anti-proliferative ability of Enz (*p* < 0.01) (Figure 3L and Table S4). Figure 3, M-O shows the results of the antimigration and invasion assays. Among all the treatments, Enz+GB4-BPL@siCXCR2/pPTEN had the greatest anti-migration and anti-invasion effects, followed by GB4-BPL@siCXCR2/pPTEN, indicating that combination therapy had a certain inhibitory effect on migration and invasion.

We also evaluated the impact of GB4-BPL@siCXCR2/pPTEN on siCXCR2 and PTEN mRNA levels in RM-1 EnzR cells (Figure 3, P and Q) and PTEN.CaP8 EnzR cells (Figure 3, R and S). The results indicated that the CXCR2 mRNA levels were significantly lower than those in both the blank and NC groups (*p* < 0.01). The PTEN mRNA levels were significantly greater than those in the blank group, the NC group (*p* < 0.01) and the Lipo8000 group (*p* < 0.05), confirming the successful delivery of siCXCR2 and pPTEN into tumor cells by GB4-BPL. These results verified the synergetic effects of siCXCR2 and pPTEN in GB4-BPL@siCXCR2/pPTEN on the inhibition of EnzR CRPC cell growth, metastasis and invasion following the knockdown of CXCR2 and the restoration of PTEN function.

To evaluate whether siCXCR2 could inhibit the EMT phenotype of CRPC cells, the protein expression levels of E-cadherin, vimentin and N-cadherin in RM-1 EnzR cells after different treatments were detected by Western blotting. As shown in Figure S19, low expression of E-cadherin and high expression of N-cadherin and vimentin were detected in untreated RM-1 EnzR cells, which exhibited a mesenchymal phenotype. After treatment with GB4-BPL@siCXCR2 and GB4-BPL@siCXCR2/pPTEN, the expression of E-cadherin increased, and the expression levels of N-cadherin and vimentin decreased, indicating the transformation of RM-1 EnzR cells to the epithelial phenotype. These results also demonstrated that siCXCR2 inhibition and PTEN restoration could inhibit EMT, ultimately leading to the suppression of tumor invasion and metastasis. Interestingly, CXCR2 is an important biomarker of neuroendocrine prostate cancer (NEPC), and our strategy is also beneficial for drug-resistant PCa therapy, as it significantly suppresses the progression of NEPC 24.

### The ability of GB4-BPL to target the tumor and bone metastatic areas of the EnzR BmCRPC

**Figure 4:**
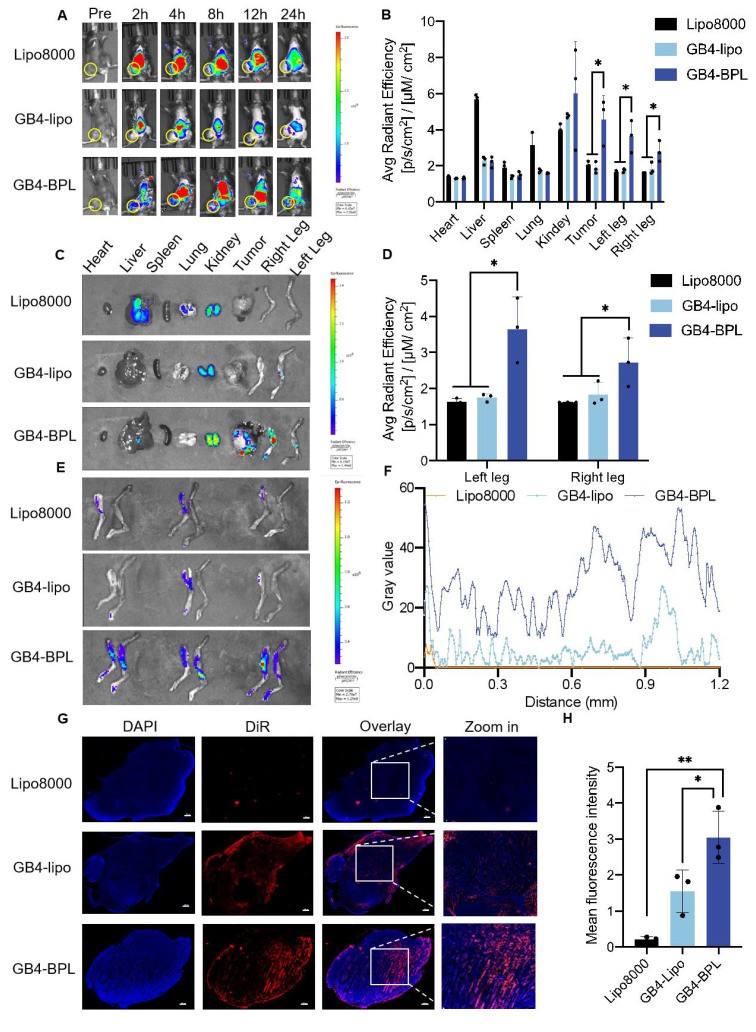
*In vivo* biodistribution of GB4-BPL. **(A)** The representative small animal living images of each group of the BmCRPC-bearing mice at 0-24 h, post-injection (yellow circle: tumor area). Lipo8000@DiR, GB4-lipo@DiR and GB4-BPL@DiR were injected at a DiR concentration of 1 mg/kg. **(B)** The qualified distribution in tumors, bones and major organs of each group. **(C)** Fluorescence imaging of tumors, bones and major organs. **(D)** Total luminescence quantification of the dissected left leg, and right leg for Lipo8000, GB4-lipo and GB4-BPL -treated mice. **(E)** Left legs, and right legs were dissected for luminescence imaging. **(F)** Image analysis of intratumor permeation of DiR-labeled Lipo8000, GB4-lipo, and GB4-BPL. **(G)** Intertumoral permeation in whole tumor mass by CLSM detections, scale bars = 500 μm. **(H)** Statistical analysis of mean fluorescence intensity for frozen tumor sections in each group. Data are mean ± SD (n = 3), *p < 0.05, **p < 0.01, ***p < 0.001, one-way ANOVA.

To evaluate the targeting abilities of GB4-BPL, we established an EnzR BmCRPC C57BL/6J mouse model in which DiR was used as a model drug. As shown in Figure 4, A-C, the fluorescence signal of the DiR-labeled GB4-BPL group could be observed at the tumor sites as early as 2 h, with a peak signal intensity obtained at 8 h post injection and maintaining a high intensity at 24 h, with reduced accumulation in the liver and lung areas. In the Lipo8000 and GB4-lipo groups, limited fluorescence signal intensity was detected in the tumor and bone areas. The high fluorescence intensity of the Lipo8000 group accumulated in the liver, lung and kidney areas, and that of the GB4-lipo group was distributed mainly in the liver and kidney areas.

Furthermore, to quantify the bone-targeting ability of GB4-BPL, bones from the hindlimbs of the mice were dissected for fluorescence analysis. The results revealed that the fluorescence intensity in the left and right legs of the GB4-BPL group was approximately twice that of the GB4-lipo group (Figure 4, D and E). Notably, the fluorescence in the GB4-lipo and Lipo8000 groups was distributed mainly at the edge of the tumor tissues without entering the interior of the tumor tissues, whereas the GB4-BPL group presented the highest fluorescence signals, with signal distributed in both the interior and exterior regions of the tumor tissue, indicating that the GB4-BPL accumulated and penetrated deeply into the bone metastatic tumor (Figure 4, F and G). The semiquantitative analysis results also revealed that the highest fluorescence intensity was obtained in the GB4-BPL group, followed by the GB4-lipo group (Figure 4H), whereas the Lipo8000 group presented the lowest signal intensity. These results demonstrated the high tumor accumulation and bone-targeting abilities of GB4-BPL, highlighting its potential for bone-targeted delivery for cancer treatment.

### Combination of pPTEN and siCXCR2 in GB4-BPL enhanced the inhibition of CRPC growth and metastasis *in vivo*

**Figure 5:**
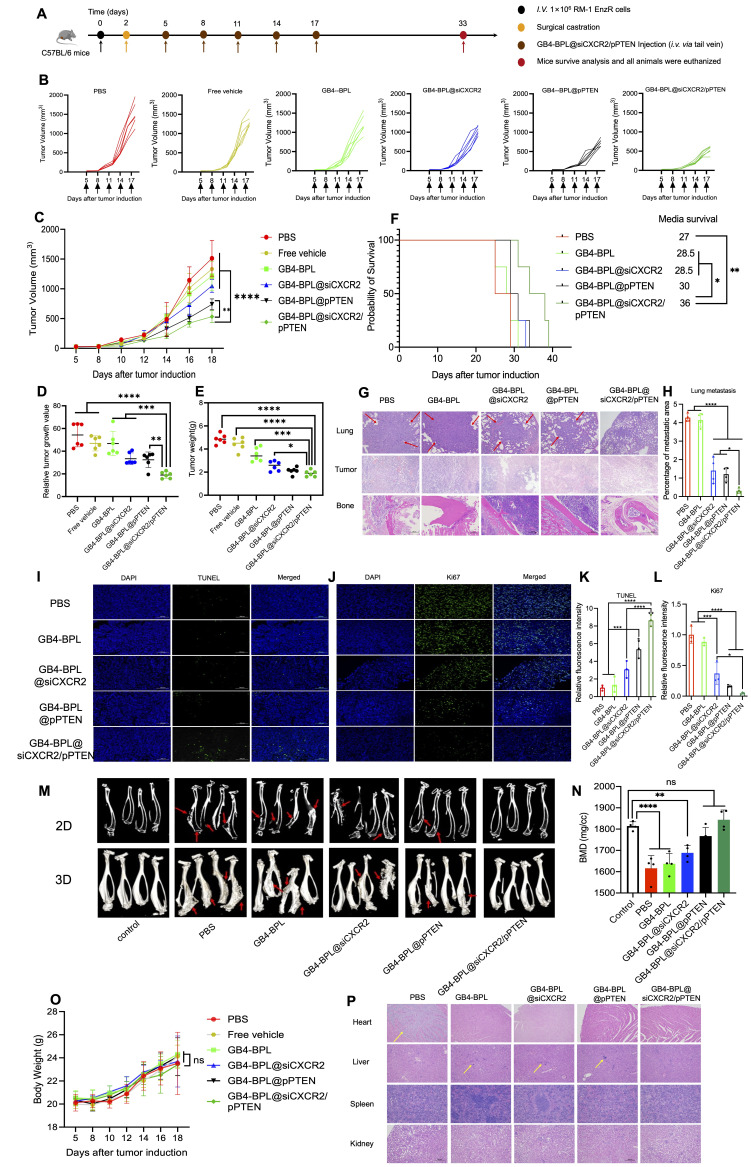
*In vivo* therapeutic effects and biosafety of GB4-BPL@siCXCR2/pPTEN in EnzR BmCRPC model.** (A)** Scheme of tumor inoculation, surgical castration and systemic injection (*i.v. via* the tail vein) of PBS, Free vehicle, GB4-BPL, GB4-BPL@siCXCR2, GB4-BPL@pPTEN, and GB4-BPL@siCXCR2/pPTEN in RM-1 EnzR tumor-bearing male C57BL/6J. Mice were castrated when the tumor volume reached 20-30 mm^3^ and received injections 3 days later. Injections were performed every 3 days for 5 times. **(B)** Individual growth curves for mice treated as indicated. **(C)** Tumor growth measurements show the *in vivo* therapeutic efficacy (n = 6). **(D)** Tumor growth index at the end time points from each treatment. **(E)** Tumor weight of the 6 groups (n = 6). **(F)** Survival curve of mice in different treatment groups (n = 4). **(G)**The HE images (200 ×) of the lung, tumor and bone of each group. Red arrow: tumor metastatic sites. **(H)** Statistical analysis of lung metastasis (n = 4).** (I)** TUNEL assay was performed to evaluate the apoptosis of tumor cells in each group. TUNEL: green, scale bar = 100 μm. **(J)** Ki67 staining (green) of tumor tissues in each group. Scale bar = 100 μm. **(K-L)** Statistical analysis of TUNEL- and Ki67-positive cells (n = 3). **(M)** The microCT images of each group in 2D and 3D (n = 4, red arrows: bone damaged sites). **(N)** The BMD values of the BmCRPC-bearing tibias of each group, tumor-free normal tibias were used as control (n = 4). **(O)** Body weight of mice growth curves (n = 6). **(P)** The HE images (100 ×) of the heart, liver, spleen, and kidney of each group. Yellow arrow: tumor inflammation site. Data are represented as means ± SD, *p < 0.05, **p < 0.01, ***p < 0.001, ****p < 0.0001, ns: no significance, one-way ANOVA.

The *in vivo* therapeutic effects and biosafety of combination therapy with pPTEN and siCXCR2 were evaluated in an EnzR BmCRPC mouse model. Figure 5A shows the scheme of tumor inoculation, surgical castration and systemic injection. Among all the treatments, GB4-BPL@siCXCR2/pPTEN exhibited greater antitumor efficacy than the GB4-BPL@pPTEN (*p* < 0.01) and GB4-BPL@siCXCR2 (*p* < 0.0001) treatments did (Figure 5, B and C). Moreover, the tumor growth index (TGI), referring to the value of tumor size at each time point compared with that at the initial time point, was only 18.6 in the GB4-BPL@siCXCR2/pPTEN group, which was much lower than those in the GB4-BPL@pPTEN (*p* < 0.01) and GB4-BPL@siCXCR2 (*p* < 0.001) groups (Figure 5D). Furthermore, the tumor weight was significantly lower in the GB4-BPL@siCXCR2/pPTEN group than in the other groups, which was consistent with the tumor volume results (Figure 5E). Interestingly, there was no significant difference in tumor weight between the GB4-BPL@pPTEN and GB4-BPL@siCXCR2/pPTEN groups (*p* > 0.05), whereas the tumor volume in the GB4-BPL@siCXCR2/pPTEN group was significantly lower than that in the GB4-BPL@pPTEN group (*p* < 0.01). The main reason for this difference might be the heterogeneity of the tumors, such as differences in necrotic regions, stromal composition, edema, and immune cell infiltration. These factors can affect tumor density and mass independent of overall volume 29,30. The survival analysis results shown in Figure 5F revealed that the GB4-BPL@siCXCR2/pPTEN group had a longer median survival time than the GB4-BPL@siCXCR2 in the Enz-resistant BmCRPC model (*p* < 0.05).

Figure 5, G and H show the results of hematoxylin‒eosin (HE) staining of the lung, tumor and bone (at the tumor site). As shown in Figure 5G, no secondary lung metastasis was detected in the GB4-BPL@siCXCR2/pPTEN group. Compared with the control treatment, GB4-BPL@siCXCR2/pPTEN significantly reduced lung metastasis (*p* < 0.0001). The combination treatment enhanced the oncolytic and bone protection effects, which further demonstrated the ability of GB4-BPL@siCXCR2/pPTEN to inhibit tumor growth with good compatibility.

*In vivo* tumor cell apoptosis was detected by TUNEL staining. As shown in Figure 5, I and K, compared with the other groups, the GB4-BPL@siCXCR2/pPTEN group presented an increased number of apoptotic cells. Notably, no TUNEL-positive cells were observed in the PBS groups. Ki67 staining of the tumor sections revealed that, compared with the other groups, the GB4-BPL@siCXCR2/pPTEN group presented a markedly diminished green fluorescent signal (Figure 5, J and L), indicating that the lowest level of Ki67-positive tumor cell proliferation was observed in the combination treatment group, which was in line with the results observed via TUNEL staining. These results demonstrated that the combination therapy synergistically delayed the progression of EnzR BmCRPC.

The degree of bone loss was subsequently evaluated by micro-CT. As shown in Figure 5M, the skeletal structure of the mice treated with GB4-BPL@siCXCR2/pPTEN maintained an intact cortical shell, whereas the other groups exhibited deformation and even fracture due to osteolysis. There was no significant difference in BMD values between the GB4-BPL@siCXCR2/pPTEN group and the control group (*p* > 0.05). In contrast, the BMD values of the other groups were significantly lower than those of the control group (*p* < 0.05), indicating severe bone loss (Figure 5N). These results demonstrated that GB4-BPL@siCXCR2/pPTEN inhibited not only the progression of CRPC growth and CRPC bone metastasis but also the secondary organ metastasis of BmCRPC.

Additionally, the H&E staining results revealed obvious cardiac inflammation in the PBS group, and liver inflammation was observed in all the groups except the GB4-BPL@siCXCR2/pPTEN group (Figure 5P), which may be due to the ability of GB4-BPL to target bone and decrease drug accumulation in the liver. In addition, the results of the biochemical analysis of mouse blood samples revealed that all indices in all groups were within their normal ranges in the Enz-resistant BmCRPC models (Figure S20). These data demonstrated the good biocompatibility of GB4-BPL@siCXCR2/pPTEN.

### GB4-BPL@siCXCR2/pPTEN enhanced Enz efficacy and significantly suppressed BmCRPC growth *in vivo*

**Figure 6:**
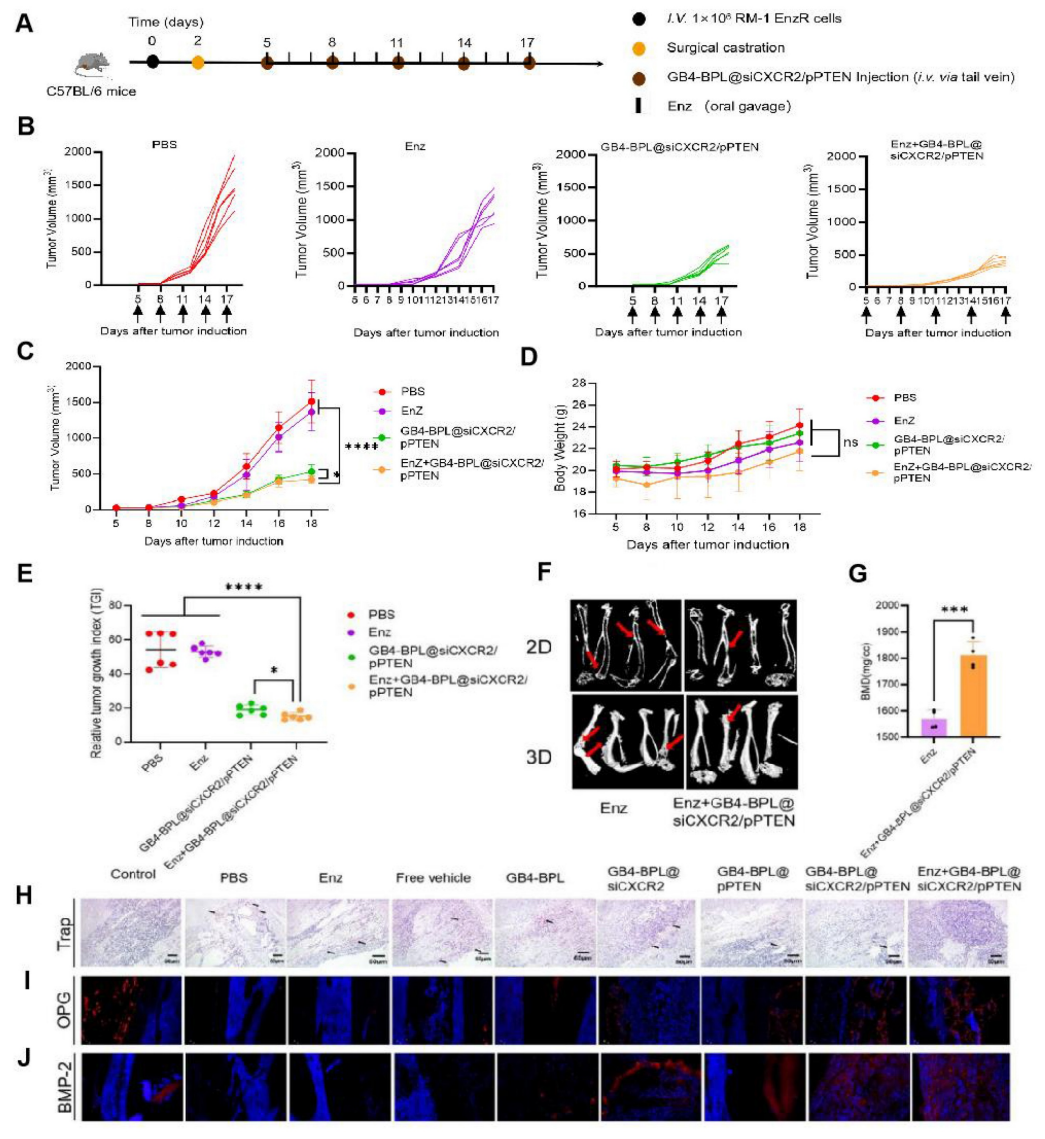
*In vivo* effects of GB4-BPL@siCXCR2/pPTEN on Enz resistance in EnzR BmCRPC model. **(A)** Scheme of tumor inoculation, surgical castration and administration of PBS, Enz, GB4-BPL@siCXCR2/pPTEN and Enz+GB4-BPL@siCXCR2/pPTEN in RM-1 EnzR tumor-bearing male C57BL/6J. Mice were castrated when the tumor volume reached 20-30 mm^3^ and received injections and oral gavage 3 days later. Injections were performed every 3 days for 5 times. Oral gavage was performed each day for 2 weeks. **(B)** Individual growth curves for mice treated as indicated (n = 6). **(C)** Tumor growth measurements show the *in vivo* therapeutic efficacy (n = 6). **(D)** Body weight of mice growth curves of the 4 groups (n = 6). **(E)** Tumor growth index at the end time points from each treatment (n = 6). **(F)** The microCT images of two groups in 2D and 3D (n = 4, red arrows: bone damaged sites). **(G)** The BMD values of the BmCRPC-bearing tibias of two groups (n = 4). **(H)** Representative Trap staining images of tumor-bearing tibias. **(I)** Representative OPG staining images of tumor-bearing tibias. **(J)** Representative BMP-2 staining images of tumor-bearing tibias. (scale bars = 20 μm). Data are represented as means ± SD, *p < 0.05, **p < 0.01, ***p < 0.001, ****p < 0.0001, ns: no significance, Student's t test and one-way ANOVA.

Figure 6 shows the *in vivo* effects of GB4-BPL@siCXCR2/pPTEN on Enz resistance in the EnzR BmCRPC model. Figure 6A shows the scheme of tumor inoculation, surgical castration and administration. As shown in Figure 6, B and C, the Enz+GB4-BPL@siCXCR2/pPTEN group presented greater antitumor efficacy than the Enz (*p* < 0.0001) and GB4-BPL@siCXCR2/pPTEN groups did (*p* < 0.05), without any obvious decrease in body weight (Figure 6D). Moreover, the TGI of the Enz+GB4-BPL@siCXCR2/pPTEN group was only 14.9, which was lower than that of the GB4-BPL@siCXCR2/pPTEN group (*p* < 0.05, Figure 6E). These results demonstrated that GB4-BPL@siCXCR2/pPTEN could reverse Enz resistance and synergistically suppress cell growth (CI < 1) (Figure S16). Moreover, the microCT results revealed that the skeletal structure of the mice treated with Enz+GB4-BPL@siCXCR2/pPTEN exhibited slight deformation (Figure 6F), and the BMD value was significantly lower than that of the Enz group (*p* < 0.001), indicating severe bone loss in the Enz group (Figure 6G). Overall, GB4-BPL@siCXCR2/pPTEN enhanced the effect of Enz and reduced its drug resistance *in vivo*.

Moreover, the TRAP staining results revealed that the PBS group had the greatest infiltration of osteoclasts, whereas the GB4-BPL@siCXCR2/pPTEN and Enz+GB4-BPL@siCXCR2/pPTEN groups had the lowest infiltration of osteoclasts, indicating the good bone-protecting effects of the treatments (Figure 6H). Following OPG staining, compared with the PBS group, the GB4-BPL@siCXCR2/pPTEN and Enz+GB4-BPL@siCXCR2/pPTEN groups presented the highest expression levels of OPG (Figure 6I). Similarly, the BMP-2 staining results also revealed that the expression levels of BMP-2 in the GB4-BPL@siCXCR2/pPTEN and Enz+GB4-BPL@siCXCR2/pPTEN groups were significantly greater than those in the PBS group (Figure 6J). Furthermore, compared with those in the GB4-BPL@siCXCR2/pPTEN group, the OPG and BMP-2 levels in the Enz+GB4-BPL@siCXCR2/pPTEN group were greater. These results fully indicated that the combination of Enz and GB4-BPL@siCXCR2/pPTEN was beneficial for bone protection and reducing bone damage.

### GB4-BPL@siCXCR2/pPTEN transformed BmCRPC from a “cold” tumor to a “hot” tumor

**Figure 7:**
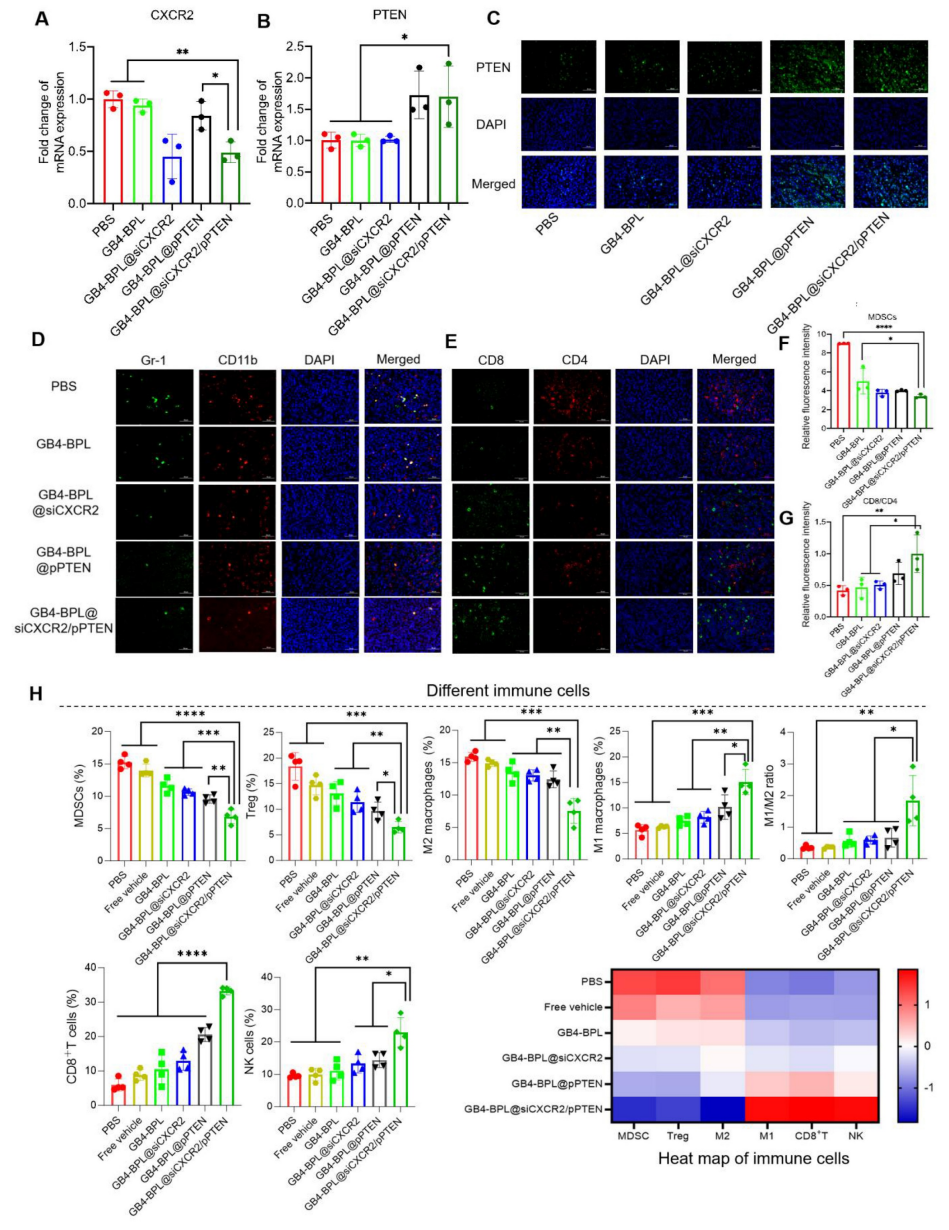
The mechanism of immune regulation. **(A, B)** The RT-qPCR results of the transcription of CXCR2 and PTEN in each group (n = 3). **(C)**IF images (400 ×) of the expression of PTEN in the tumor site of each group. **(D)** Representative images of MDSCs (CD11b^+^Gr-1^+^) in tumor of mice in different treatment groups. **(E)** Representative images of CD8^+^ T cells and CD4^+^ T cells in tumor of mice in different treatment groups.** (F-G)** Statistical analysis of CD11b^+^Gr-1^+^MDSCs and CD8^+^/CD4^+^ ratio (n = 3). **(H)** The frequency or percentage of different immune cells including MDSCs or NK cells (the proportion in total tumor cells), Tregs or CD8^+^ T cells (the proportion in CD45^+^CD3^+^ cell population), M2- or M1-macrophages (the proportion in the CD11b^+^F4/80^+^ cell population) as well as the summarized heat map of immune cells in tumors from each treatment. (n = 4, mean ± SD). *p < 0.05, **p < 0.01, ***p < 0.001, ****p < 0.0001, one-way ANOVA.

Next, to investigate the mechanisms of GB4-BPL@siCXCR2/pPTEN, the mRNA expression levels of PTEN and CXCR2 in tumor tissues were detected by RT‒qPCR. As shown in Figure 7A, an approximately 50% decrease in CXCR2 transcription was detected in the GB4-BPL@siCXCR2/pPTEN group, which was considerably greater than those in the other groups (*p* < 0.05). Moreover, the transcription of *Pten* was increased compared with those in the PBS, GB4-BPL, and GB4-BPL@siCXCR2 groups (Figure 7B). Additionally, immunofluorescence (IF) images revealed a more intuitive increase in PTEN (Figure 7C).

Bone marrow cell differentiation and functional abnormalities are critical hallmarks of cancer 31. As key immunosuppressive cells derived from the bone marrow, MDSCs impair T-cell activation during tumor development 32. To determine whether PTEN restoration and CXCR2 depletion could reactivate antitumor immunity by remodulating the immune microenvironment of BmCRPCs, we analyzed MDSC (CD11b^+^Gr-1^+^) infiltration in tumor tissues after 2 weeks of treatment. Notably, compared with the other groups, the GB4-BPL@siCXCR2/pPTEN group presented the lowest proportion of MDSCs (Figure 7, D and F). Moreover, CD4^+^ T cells play a regulatory role in immune responses, and CD8^+^ T cells serve as vital effector cells in the context of antitumor responses, with an elevated CD8^+^ T-cell ratio indicating a more potent antitumor effect 33. Our study demonstrated that GB4-BPL@siCXCR2/pPTEN increased CD8^+^ T-cell infiltration while reducing CD4^+^ T-cell proportions. Notably, this treatment resulted in the highest CD8^+^/CD4^+^ T-cell ratio among all the treatment groups (Figure 7, E and G), suggesting a more potent antitumor immune response compared with the other treatments.

Moreover, the frequencies of different immune cells in tumor tissues were measured by flow cytometry (Figure 7H). The results revealed that the lowest proportions of immunosuppressive cellular components, such as MDSCs, Tregs, and M2-like TAMs, were found in the GB4-BPL@siCXCR2/pPTEN group. Compared with those in the PBS group, the frequencies of MDSCs, Tregs, and M2-like TAMs in the GB4-BPL@siCXCR2/pPTEN-treated tumors were reduced by 55.01%, 64.75%, and 52.53%, respectively. Moreover, the proportions of these immunosuppressive cells in the GB4-BPL@siCXCR2/pPTEN-treated tumors were significantly lower than those in the GB4-BPL@siCXCR2- and GB4-BPL@pPTEN-treated tumors (*p* < 0.05)—MDSCs were reduced by 35.68% and 28.33%, Tregs by 43.15% and 32.82%, and M2-like TAMs by 42.05% and 39.26%, respectively. Furthermore, the effects of GB4-BPL@siCXCR2/pPTEN on antitumoral contributors to cancer immunotherapy, including M1-like macrophages, NK cells, and CD8^+^ T cells, were also evaluated. The results revealed that the GB4-BPL@siCXCR2/pPTEN group had the highest percentage of M1 macrophages, at 1.84- and 1.48-fold greater than those in the GB4-BPL@siCXCR2 and GB4-BPL@pPTEN groups, respectively. Consequently, the ratio of antitumoral M1 macrophages to protumoral M2 phenotypes (M1/M2 ratio) in GB4-BPL@siCXCR2/pPTEN-treated tumors was 3.10-fold greater than that in the GB4-BPL@siCXCR2 group, and 2.77-fold greater than that in the GB4-BPL@pPTEN group, suggesting the notable M2‒M1 repolarization effect of GB4-BPL@siCXCR2/pPTEN. Moreover, the frequency of NK cells in GB4-BPL@siCXCR2/pPTEN-treated tumors was significantly greater than those in the other groups (*p* < 0.05). Importantly, the population of CD3^+^CD8^+^ T cells in the GB4-BPL@siCXCR2/pPTEN group was the highest, at 2.57- and 1.62-fold greater than those in the GB4-BPL@siCXCR2 and GB4-BPL@pPTEN groups, respectively. As a result, GB4-BPL@siCXCR2/pPTEN treatment increased the M1/M2 ratio of macrophages, the proportion of CD3^+^CD8^+^ T lymphocytes, and the frequency of NK cells in tumor tissues, highlighting the great potential of GB4-BPL@siCXCR2/pPTEN to increase antitumor immunity (Figure 7H, S21-S25). This heightened immune response was a pivotal contributor to the effective inhibition of CRPC development in the combination therapy group.

## Discussion

Patients with CRPC eventually become resistant to Enz via multiple mechanisms 5,34 and progress to metastatic CRPC (mCRPC), with nearly 90% of cases metastasizing to the bone, leading to serious skeletal-related events (SREs), including bone injury, bone loss, bone pain and osteoporosis, which can significantly reduce the quality of life of affected patients. Moreover, more than 49% of patients with mCRPC have functional loss of *PTEN*, promoting AR inhibitor resistance 11. Loss of *PTEN* with subsequent activation of Akt is correlated with a high Gleason score and a high number of MDSCs and lower CD8^+^ T-cell abundance in CRPC bone metastasis, especially in *PTEN*-null PCa 13,14, and is greater than that of other infiltrating immune cell subsets in the mCRPC microenvironment, especially in *PTEN* null PCa 15-17. Restoration of PTEN function can not only prevent Enz resistance and bone metastasis but also reverse the immunosuppressive TME to achieve significant inhibition of tumor growth 18,19,35. In addition, immunosuppressive cytokines such as IL-23, IL-8 and CXCL5, which are related to MDSCs, promote CRPC metastasis and Enz resistance. Therefore, the combination of siCXCR2 and pPTEN could be a potential strategy for increasing Enz sensitivity and delaying the progression of CRPC metastasis. However, delivery of these two gene drugs to metastatic sites such as bone with high transfection and low toxicity is a challenge.

In this study, we synthesized a new ionizable lipid, GB4-BPL, which was further mixed with β-sitosterol, phospholipids and PEG-lipids to form lipid nanoparticles for the codelivery of siCXCR2 and pPTEN. GB4-BPL was synthesized based on pamidronate, a bisphosphonate drug that can adhere to hydroxyapatite binding sites on bone surfaces, especially on bone surfaces that are undergoing active bone resorption, making it suitable for the development of bone-targeted nanocarriers 36-38. GB4-BPL also exhibits tumor-targeting ability due to its enhanced permeability and retention (EPR) effect. Our studies revealed that these pamidronate-based lipid nanoparticles (GB4-BPL) had similar gene transfection ability to that of Lipo8000, with greater safety (Figure 1, E, F and H). Moreover, the results of the cellular uptake and 3D tumor sphere penetration tests revealed that, compared with unmodified GB4-lipo and Lipo8000 controls, GB4-BPL had greater cellular uptake by BmCRPC spheroids (Figure 2, A, B and F), indicating good gene transfection and bone-targeting abilities. Additionally, the *in vivo* biodistribution study further demonstrated that, compared with GB4-lipo (without pamidronate modification) and Lipo8000, GB4-BPL (with pamidronate) had dual tumor and bone-targeting ability and tumor drug accumulation as well as long-term circulation ability (Figure 4). These results demonstrated that GB4-BPL is an ideal vector for tumor- and bone-targeted delivery of gene drugs with high transfection efficiency and good biocompatibility. Furthermore, codelivery of pPTEN and siCXCR2 in these lipid nanoparticles substantiated the increased effective growth inhibition of EnzR BmCRPC cells, particularly in *Pten*-deficient drug-resistant cells, *in vitro* (Figure 3, A-D). The *in vivo r*esults also demonstrated that GB4-BPL@siCXCR2/pPTEN had the highest efficacy in impeding tumor progression and metastasis and improving survival with low toxicity (Figure 5, B, C, E and F), enhancing the EnzR BmCRPC model after castration. In addition, the bone microenvironment is suitable for the survival of tumor cells to acquire new characteristics, which then lead to drug resistance, immune escape, tumor recurrence, and secondary organ metastasis 39. In this study, secondary metastases from the bone to the lung were observed in all the groups except the GB4-BPL@siCXCR2/pPTEN group (Figure 5, G, H, M and N), which demonstrated that the combination of siCXCR2 and pPTEN could remodel the immunosuppressive tumor microenvironment. The IF staining and RT‒qPCR results further confirmed that after treatment, CD8^+^ T-cell infiltration into tumors increased, whereas the numbers of CD4^+^ T cells and MDSCs decreased, possibly due to remodeling of PTEN expression and a reduction in CXCR2 expression (Figure 7, A, B, D and E). Similar results have shown that a CXCR2 inhibitor in combination with Enz can effectively treat AR inhibitor-resistant patients with mCRPC by reducing the intratumor infiltration of MDSCs 25. Importantly, micro-CT assays further verified that this combined therapeutic strategy could be used to treat BmCRPC by alleviating the burdens of SREs (Figure 5, M and N), resulting in less bone damage and secondary organ metastases, providing a good idea for the treatment of BmCRPC and drug-resistant CRPC.

Previous studies have fully recognized the key negative roles of MDSCs and TAMs in the progression and immune escape of prostate cancer 40. Our study revealed that GB4-BPL@siCXCR2/pPTEN can reduce the frequencies of MDSCs, Tregs and M2-like TAMs, suggesting the possibility of preventing immune escape. Moreover, GB4-BPL@siCXCR2/pPTEN increased the frequencies of M1-like macrophages, NK cells, and CD8^+^ T cells, which is essential for enhancing the body's antitumor immune response (Figure 7H). Furthermore, we explored whether GB4-BPL@siCXCR2/pPTEN could reverse Enz resistance. The *in vitro* and *in vivo* results showed that GB4-BPL@siCXCR2/pPTEN enhanced the antitumor effects of Enz, including promoting tumor cell apoptosis; inhibiting tumor cell proliferation, invasion and metastasis; and successfully reversing Enz resistance (Figure 3, E, F and K-O and Figure 6, B and C). Enz has been reported to increase the risk of fracture in patients and to have a certain effect on bone damage 41. Our study revealed that GB4-BPL@siCXCR2/pPTEN can increase BMD and has a protective effect on bone (Figure 6, F and G). PCa is prone to metastasize to bone sites, causing osteolytic lesions and leading to imbalanced bone metabolism, increased osteoclast activity, and intensified bone resorption. Our study revealed that Enz+GB4-BPL@siCXCR2/pPTEN significantly increased OPG and BMP-2 expression (Figure 6, I and J). On the one hand, OPG competes with RANKL to block signaling pathways, inhibit osteoclast differentiation and activity, and reduce bone resorption. On the other hand, BMP-2 induces the differentiation of mesenchymal cells into osteoblasts and accelerates their proliferation. By increasing the expression of OPG and BMP-2, GB4-BPL@siCXCR2/pPTEN could enhance the ability of Enz to inhibit bone resorption, promote bone formation, reduce tibial osteolytic lesions in prostate cancer and enhance bone protection.

## Conclusions

In this study, we designed pamidronate-linked bone-targeted lipid nanoparticles (GB4-BPLs) for codelivery of pPTEN and siCXCR2, which could accurately reach bone metastatic sites with deep tumor penetration capacity and high transfection ability and biocompatibility. Moreover, siCXCR2 in combination with pPTEN in GB4-BPL overcame drug resistance and inhibited tumor growth, bone metastasis and secondary organ metastasis with less bone damage by restoring PTEN function and regulating the tumor immune microenvironment. Additionally, GB4-BPL@siCXCR2/pPTEN enhanced the efficacy of Enz in drug-resistant models. Therefore, this combination therapeutic strategy based on bone-targeted lipid nanoparticles expands the treatment options for drug-resistant BmCRPC.

## Supplementary Material

Supplementary figures and table.

## Abbreviations

Prostate cancer: PCa
ADT: Androgen deprivation therapy
CRPC: Castration-resistant prostate cancer
PTEN: Phosphatase and tensin homologue
Enz: Enzalutamide
BmCRPC: Bone metastatic castration-resistant prostate cancer
CXCR2: C-X-C motif chemokine receptor 2
IL-23: Interleukin-23
BP: Bisphosphonate
pPTEN: PTEN plasmid DNA
NEPC: Neuroendocrine prostate cancer
