# Supplementary material for: PTEN restoration and CXCR2 depletion synergistically enhance the effect of enzalutamide and inhibit bone metastatic CRPC
Source: Theranostics. 2025 Jul 25;15(16):8488–508. doi: 10.7150/thno.114534 (PMC12374621; doi:10.7150/thno.114534)
Supplement: Supplementary file 1 — Supplementary figures and table. [file thnov15p8488s1.pdf]

Supplementary Results

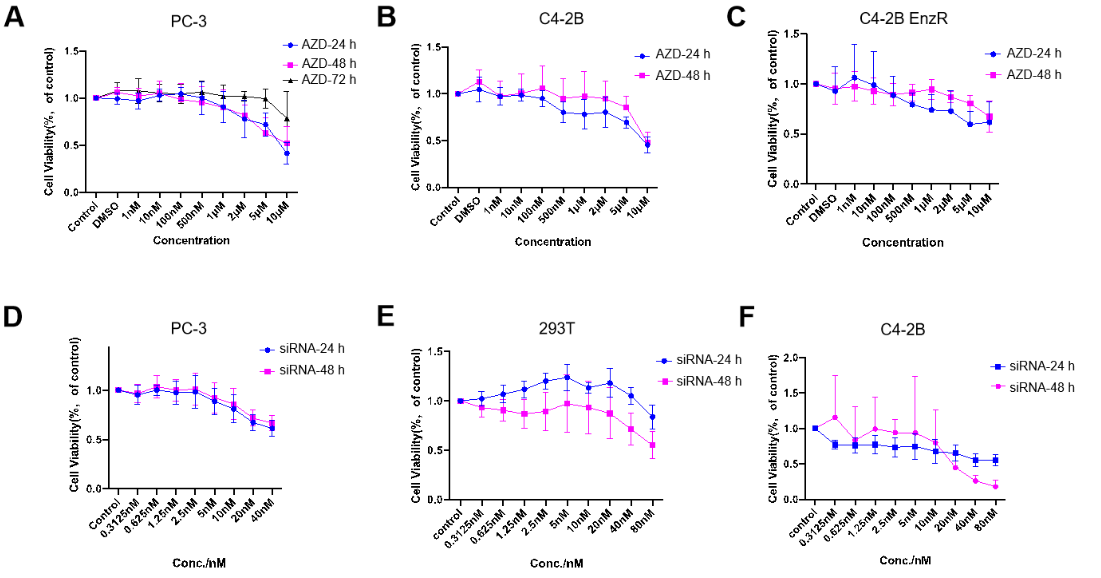

Figure S1. The cytotoxicity results of CXCR2 small molecule inhibitor (AZD5069) and CXCR2 siRNA. (A-C) PC-3, C4-2B and C4-2B EnzR cells were treated with AZD5069 for 24 h and 48 h (AZD5069: 1 nM – 10 μM). Among them, PC-3 cells investigated the cytotoxicity after 72 h. (D-F) PC-3, 293T and C4-2B cells were treated with CXCR2 siRNA for 24 h and 48 h (CXCR2 siRNA: 0.3125 nM – 80 nM).

Table S1 The IC_{50} values of AZD5069 and CXCR2 siRNA on each cell lines at 24 h and 48 h.

AZD5069

Time	IC50		
	PC-3	C4-2B	C4-2B EnzR
24 h	10.10 μM	12.37 μM	39.73 μM
48 h	8.364 μM	9.996 μM	27.15 μM

siRNA

Time	IC50		
	PC-3	293T	C4-2B
24 h	63.35 nM	~	247.8 nM
48 h	58.84 nM	116.3 nM	21.07 nM

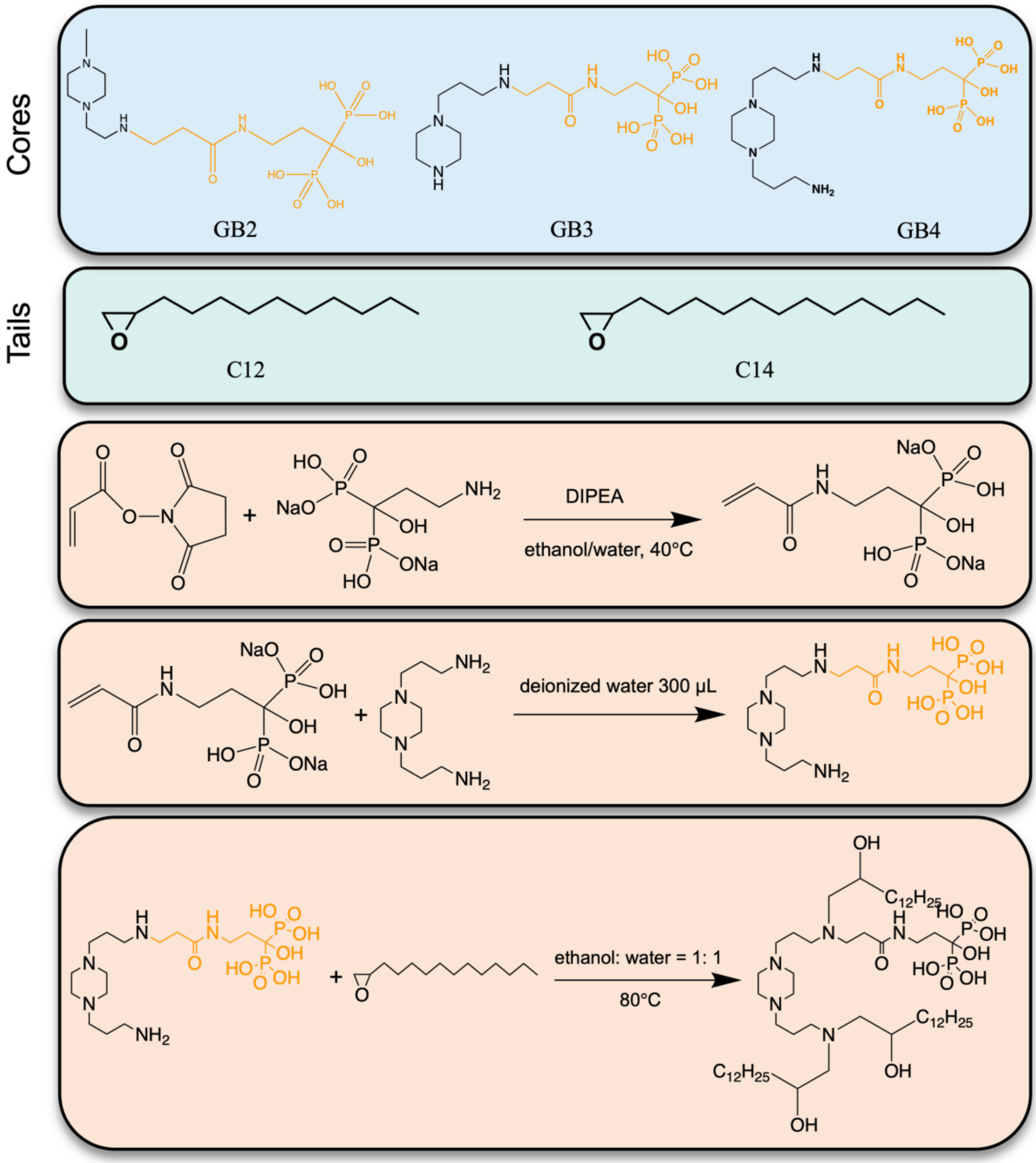

Figure S2. The synthesis steps of GB4-C14.

Compound 1. To a flame-dried 50 mL round-bottom flask charged with acryloxysuccinimide (NAS) (101.48 mg, 0.6 mmol, 1.2 equiv), pamidronate sodium (135 mg, 0.5 mmol, 1.0 equiv), and di-isopropylethylamine (DIPEA, 129.24 mg, 1.0 mmol, 2.0 equiv), was added 2 mL deionized water and ethanol respectively. The reaction mixture was heated to 40°C overnight before being cooled to ambient temperature. The solvent was removed by vacuum distillation to obtain the crude product, which was then slurried with dichloromethane and filtered to obtain a solid. Then water was added to the solid until it is completely dissolved, and then acetone was slowly added to precipitate crystals. The final product was obtained by recrystallization and could be used without further purification. ^{1}H NMR (400MHz, D2O) δ (ppm): 6.248-6.09 (m, J = 15.8, 2H), 5.705-5.68 (d, J = 10, 1H), 3.559-3.521 (t, J = 7.6, 2H), 2.177-2.159 (t, J = 7.2, 2H). LC-MS (m/z): Calculated for $[M+H]^-$: 289.11, Found: 288.23.

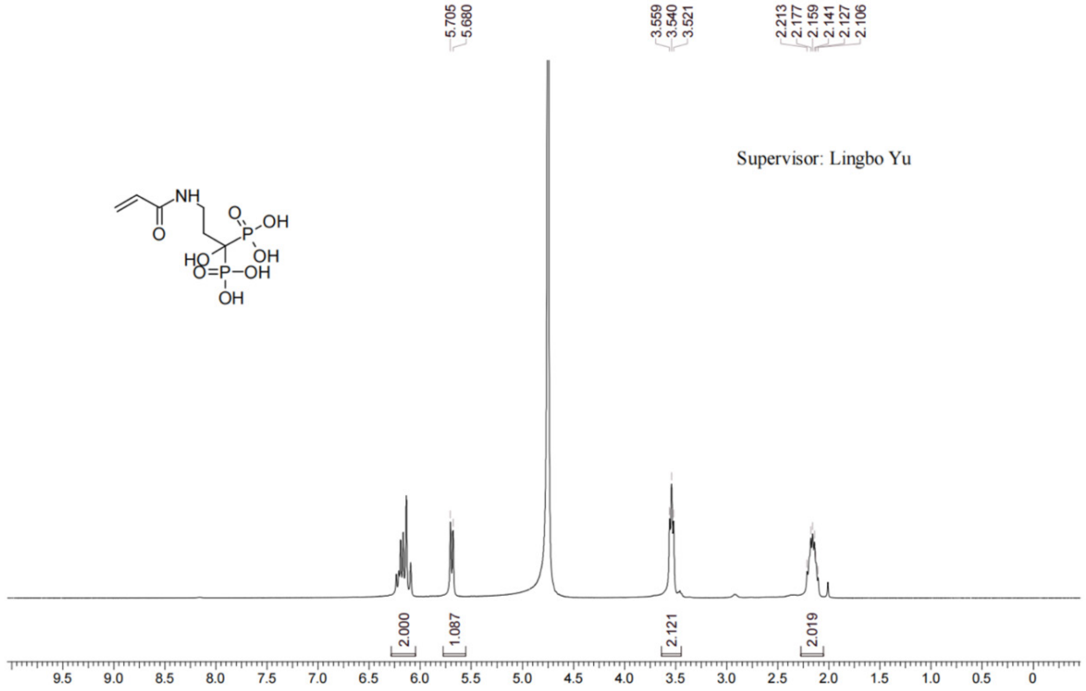

Figure S3. Characterization of Compound 1 by ^{1}H NMR spectrum.

LC-MS (m/z): Calculated for $[M+H]^-$: 289.11, Found: 288.23.

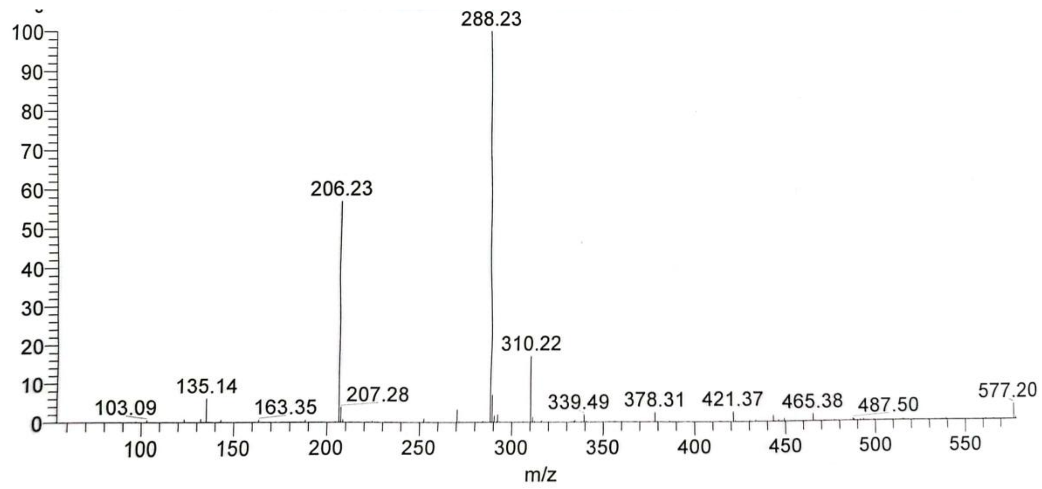

Figure S4. LC-MS analysis of Compound 1.

Compound 2. To a glass vial charged with pamidronate acrylamine (100 mg, 0.307 mmol, 1 equiv) and 1,4-bis(3-aminopropyl) piperazine (224.8 mg, 1.538 mmol, 5 equiv), was added deionized water (300 μL). The reaction mixture was conducted 40℃ overnight at room temperature. Then water was added to the solvent until it was completely dissolved, and then acetone was slowly added to precipitate crystals. The final product was obtained by recrystallization and could be used without further purification. ^{1}H NMR (400MHz, D_2O) δ (ppm): 3.416-3.378 (t, J = 15.2, 2H), 2.928-2.638 (m, J = 7.73, 8H), 2.368-2.31 (m, J = 5.8, 12H), 1.639-1.605 (m, J = 4.53, 5H). LC-MS (m/z): Calculated for $[M+H]^+$: 489.44, Found: 490.32.

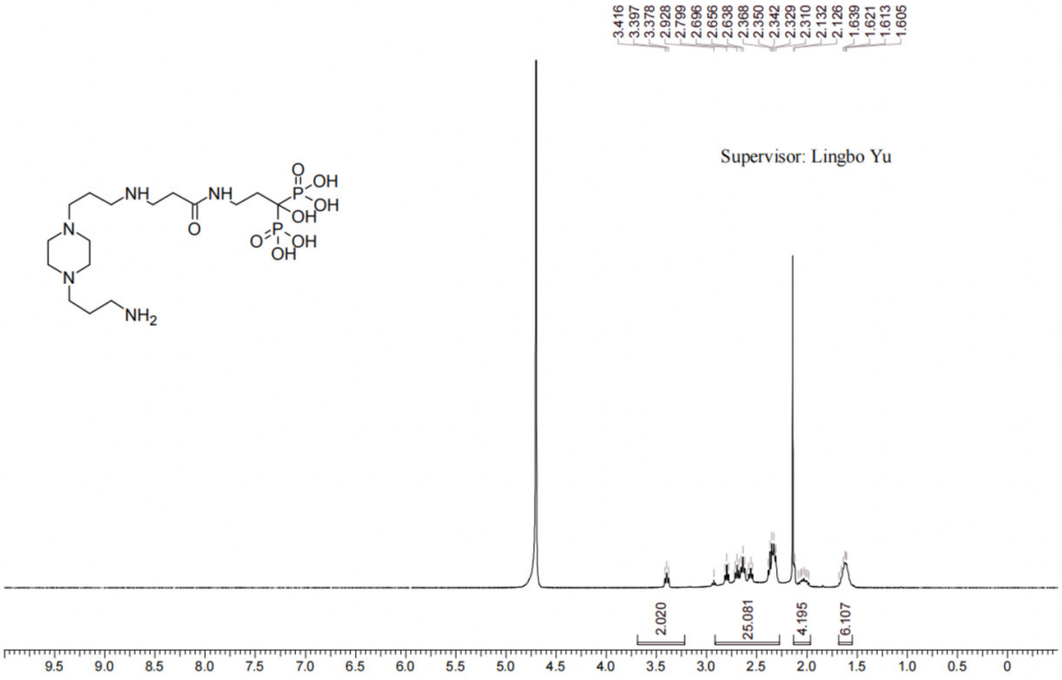

Figure S5. Characterization of Compound 2 by ^{1}H NMR spectrum.

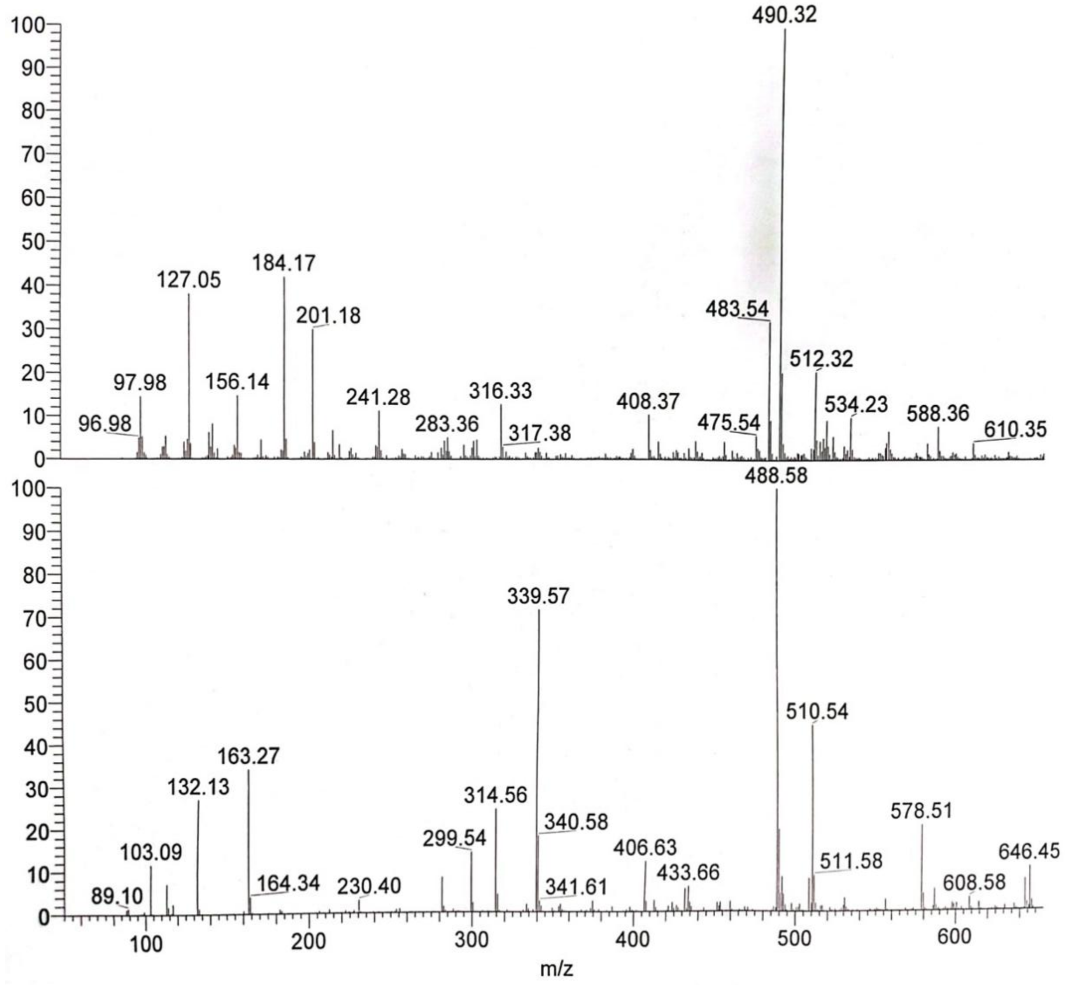

Figure S6. LC-MS analysis of Compound 2.

Compound 3. To a glass vial charged with compound 2 (9.85 mg, 0.015 mmol, 1 equiv) and C14 (15.29 mg, 0.072 mmol, 4.8 equiv), was added 2 mL deionized water and ethanol respectively. The reaction mixture was heated to 80℃ for three days before being cooled to ambient temperature. Then water was added to the solvent until it was completely dissolved, and then acetone was slowly added to precipitate crystals. The final product was obtained by recrystallization and could be used without further purification. ^{1}H NMR (400 MHz, D_2O) δ (ppm): 3.633-3.580 (m, J = 7.07, 5H), 2.840-2.454 (m, J = 6.18, 18H), 1.501-1.436 (m, J = 4.33, 70H), 1.296-1.161 (m, J = 7.71, 8H). LC-MS (m/z): Calculated for $[M+H]^+$: 1126.6, Found: 1127.0.

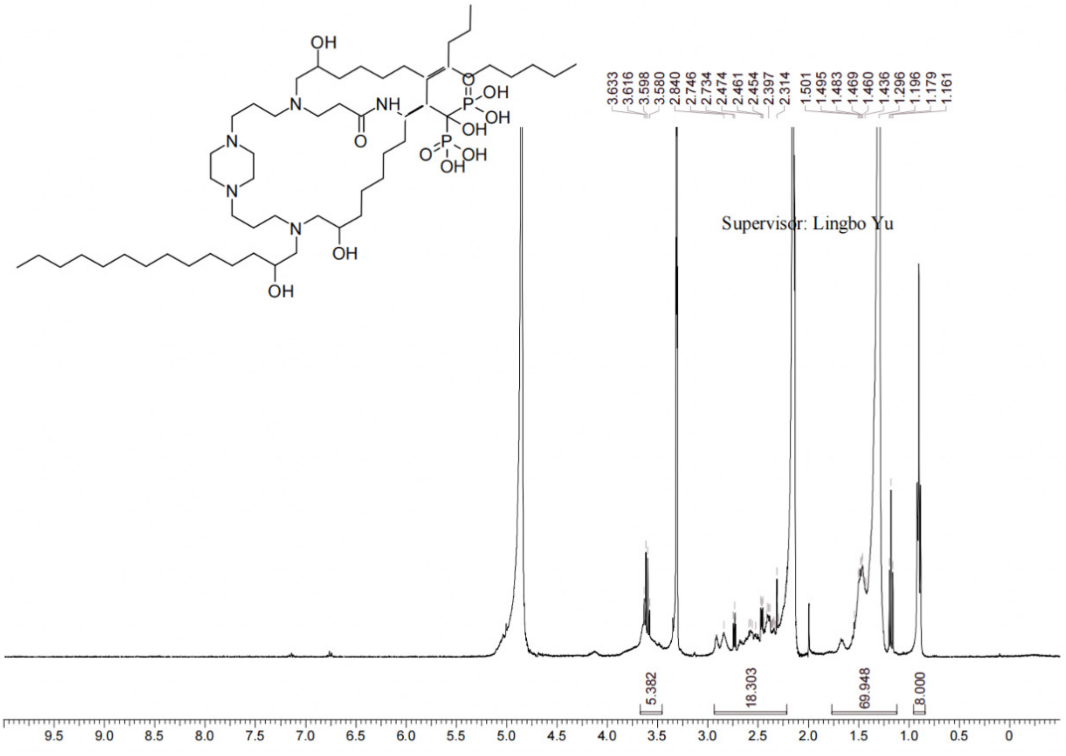

Figure S7. Characterization of Compound 3 by ^{1}H NMR spectrum.

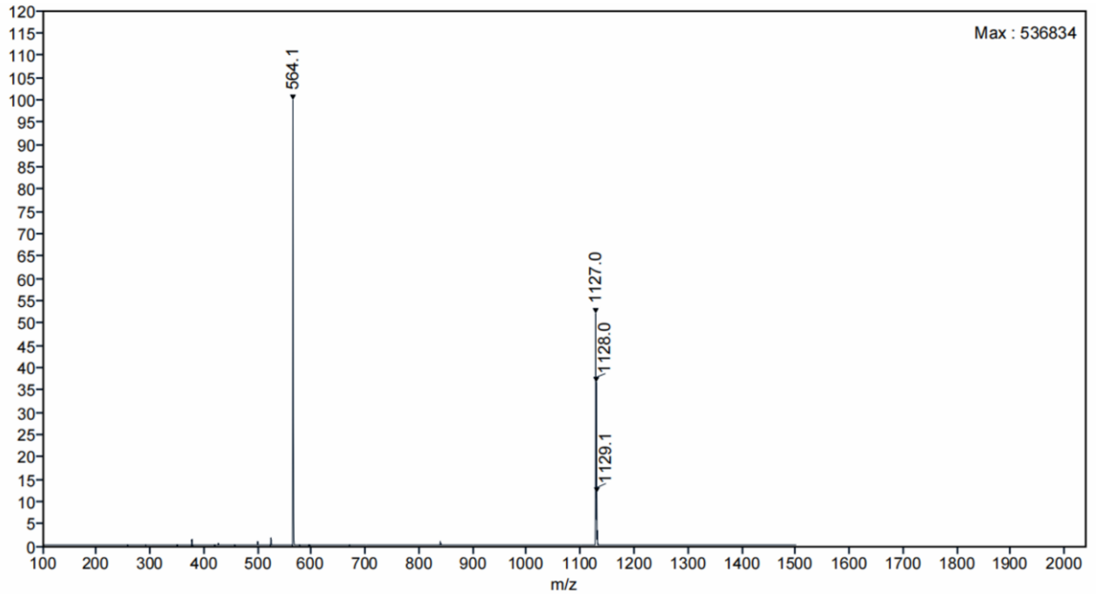

Figure S8. LC-MS analysis of Compound 3.

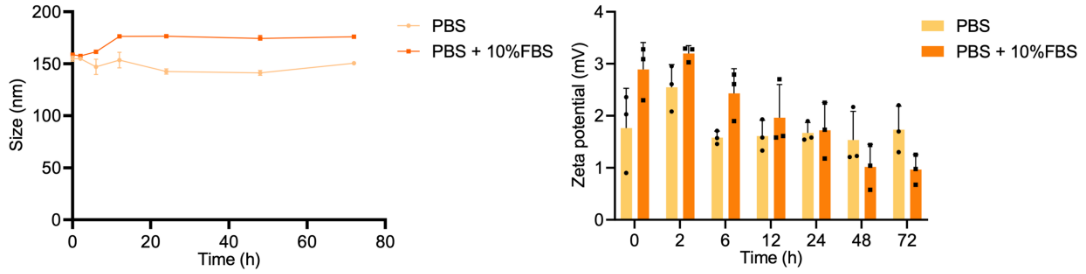

Figure S9. The stability of GB4-BPL in PBS (pH 7.4) or PBS (pH 7.4) containing 10% FBS during 72 h incubation (n = 3, mean ± SD).

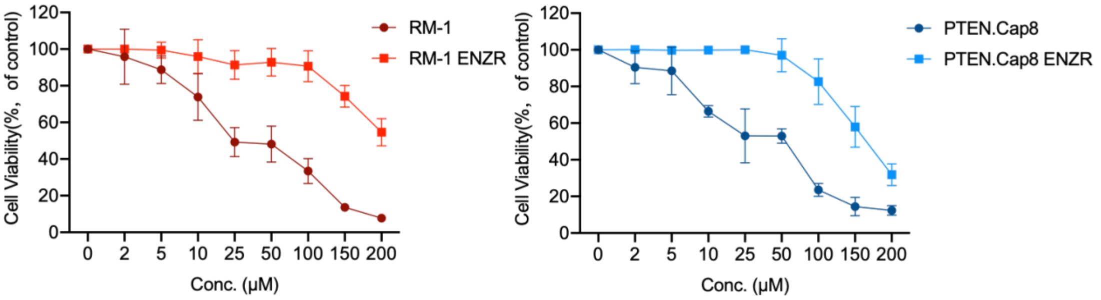

Figure S10. The CCK-8 cytotoxicity test results of enzalutamide for co-incubating 48 h with RM-1, RM-1 EnzR, PTEN.CaP8 and PTEN.CaP8 EnzR cells, the concentration gradients of enzalutamide were 2, 5, 10, 25, 50, 100, 150 and 200 μM (n = 3, mean ± SD).

Table S2. The IC_{50} values of enzalutamide (Enz) for each cell lines.

Group	RM-1	RM-1 EnzR	PTEN.CaP8	PTEN.CaP8 EnzR
IC50 (μM)	33.04	218.9	31.43	161.9

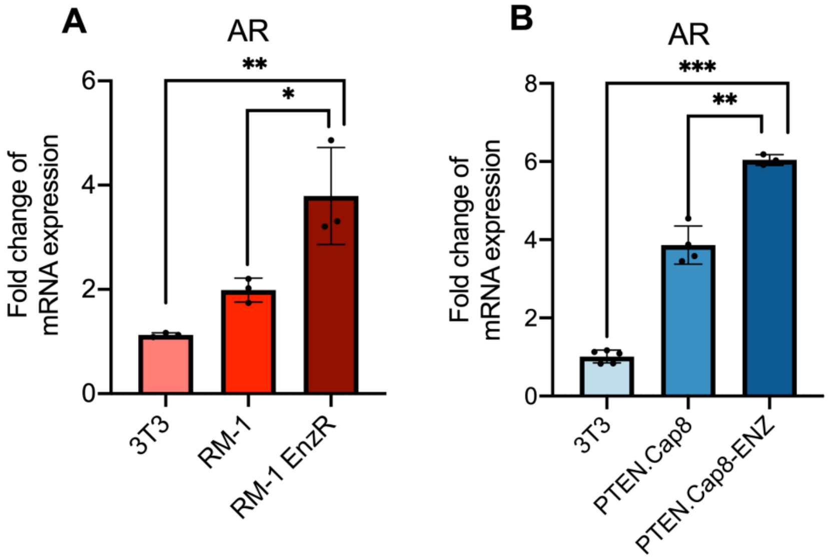

Figure S11. The RT-qPCR results of the expression level of AR mRNA in (A) RM-1 and RM-1 EnzR cells, (B) PTEN.CaP8 and PTEN.CaP8 EnzR cells.

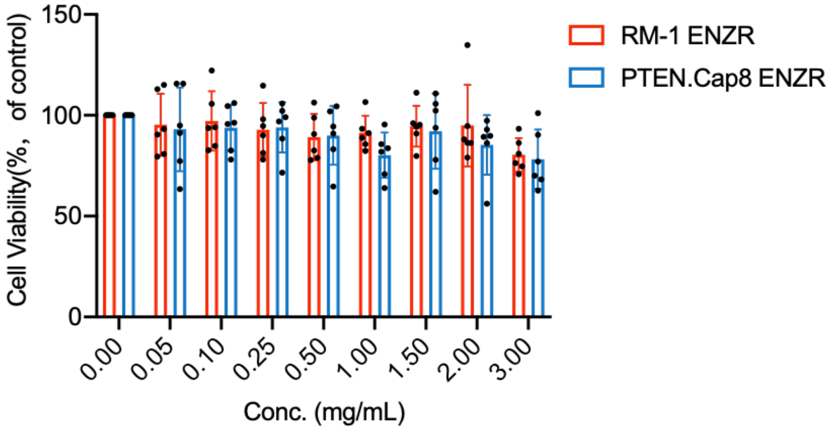

Figure S12. The cytotoxicity of GB4-BPL carriers in RM-1 EnzR, and PTEN.CaP8 EnzR cells. (GB4-BPL: 0~3 mg/mL, n = 6, mean ± SD).

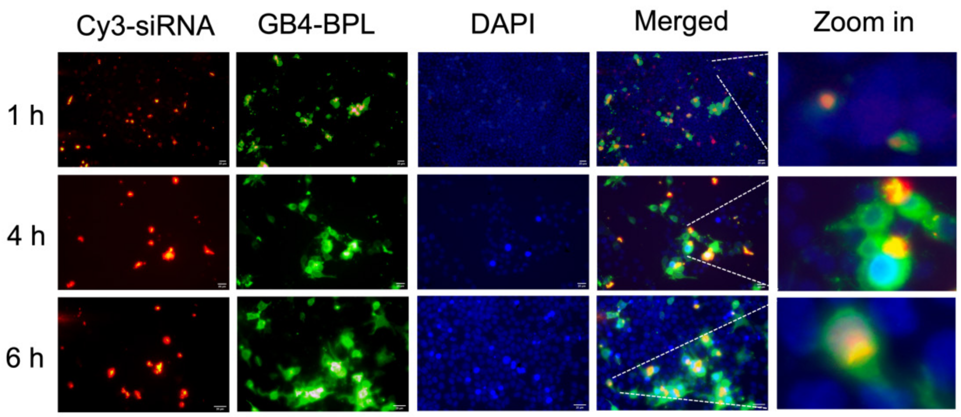

Figure S13. Cellular uptake of GB4-BPL@Cy3-siRNA, witnessed with a fluorescent microscope at 1 h, 4 h, and 6 h (Cy3-siRNA: 20 nM, scale bars = 20 μm).

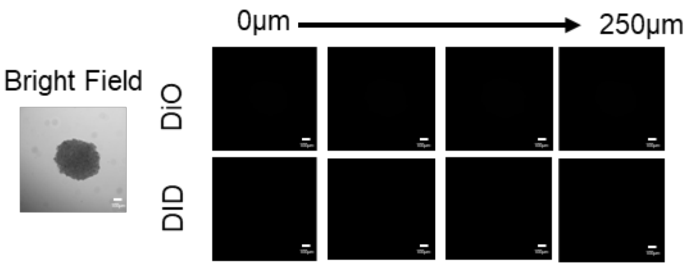

Figure S14. 3D tumor spheroid model. No-stained RM-1 EnzR cells and MC3T3 cells were seeded in the 96-well plates at the ratio of 1:1. Green fluorescence channel: λex/λem = 484 nm/501 nm (DiO), red fluorescence channel : λex/λem = 644 nm/655 nm (DiD), scale bars = 100 μm.

Table S3. The IC_{50} values of each group in RM-1 EnzR cells , PTEN.CaP8 EnzR cells and 22RV1 cells.

Group	RM-1 EnzR	PTEN.CaP8 EnzR	22RV1
NC	NS*	NS	NS
siCXCR2	147.6 nM	177.0 nM	NS
pPTEN	2744 μg/mL	108.6 μg/mL	/
GB4-BPL@siCXCR2	278.0 nM	58.84 nM	64.53 nM
GB4-BPL@pPTEN	2.849 μg/mL	0.611 μg/mL	/
GB4-BPL@siCXCR2/pPTEN	8.3nM; 1.156 μg/mL	2.224nM; 0.4321 μg/mL	/
Enz	/	/	63.28 μg/mL
Enz+GB4-BPL@siCXCR2/pPTEN	/	/	28.38 μg/mL; 4.657 nM

*NS： No significance. Data expressed in nM represents the IC_{50} value of siCXCR2, while data expressed in μg/mL represents the IC_{50} value of pPTEN and Enz.

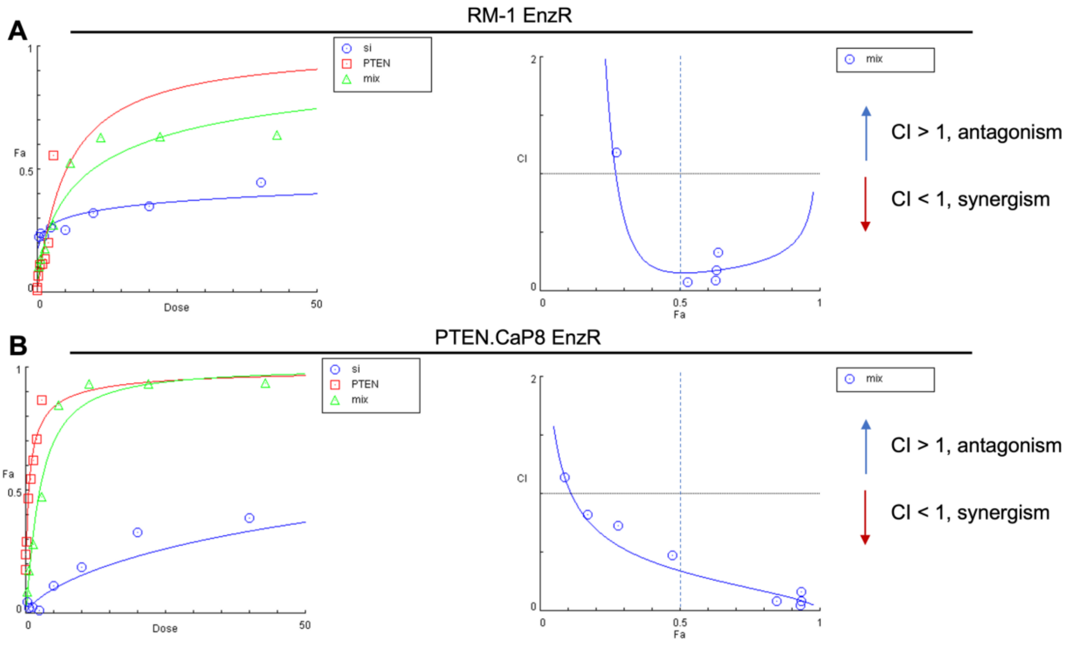

Figure S15. Combination index (CI) of pPTEN and siCXCR2 in (A) RM-1 EnzR and (B) PTEN.CaP8 EnzR cells after 48 h treatment.

Table S4. The IC_{50} values of each group in RM-1 EnzR cells.

Group	RM-1 EnzR
NC	NS
Enz	140.3 μg/mL
GB4-BPL@siCXCR2/pPTEN	8.772 nM; 1.182 μg/mL
Enz+GB4-BPL@siCXCR2/pPTEN	28.32 μg/mL; 4.351 nM; 0.708 μg/mL

Note: From left to right are the IC_{50} values of Enz, siCXCR2 and pPTEN.

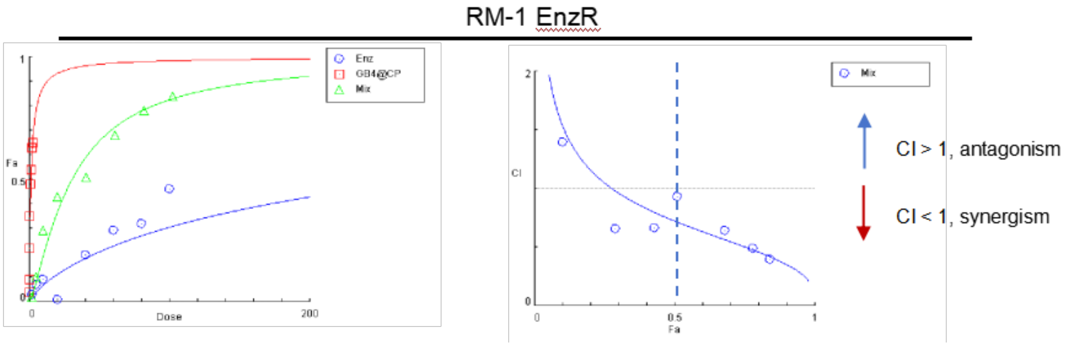

Figure S16. Combination index (CI) of Enz and GB4-BPL@siCXCR2/pPTEN in RM-1 EnzR cells after 48 h treatment.

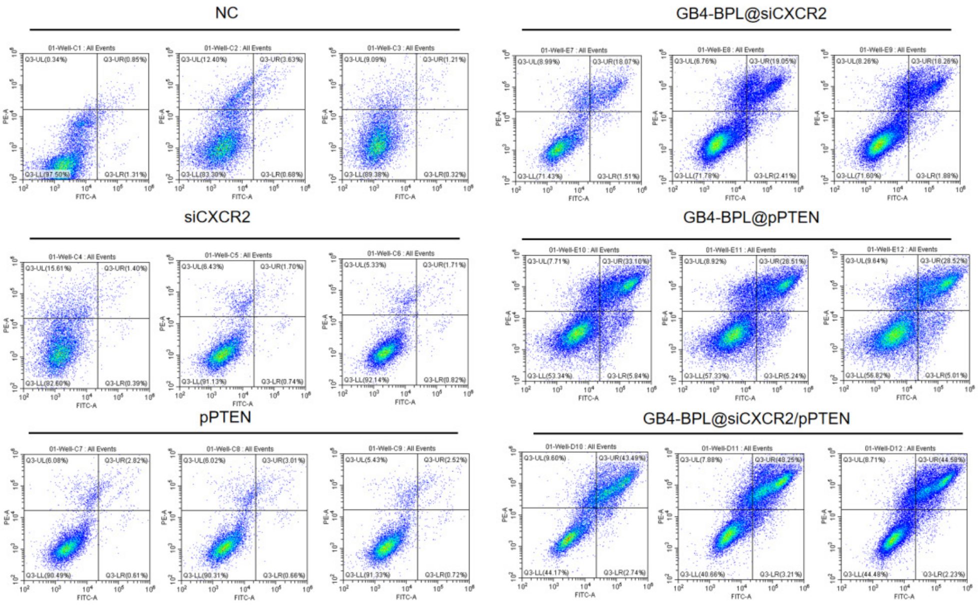

Figure S17. The representative results of apoptosis of RM-1 EnzR cells treated with each group for 48 h (siCXCR2: 8.3 nM, pPTEN: 1.156 μg/mL).

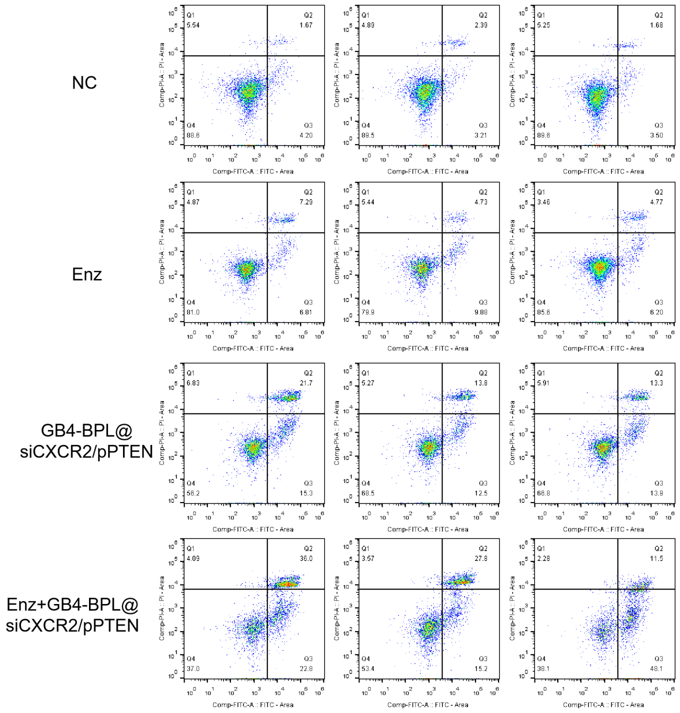

Figure S18. The representative results of apoptosis of RM-1 EnzR cells treated with each group for 48 h (siCXCR2: 8.3 nM, pPTEN: 1.156 μg/mL).

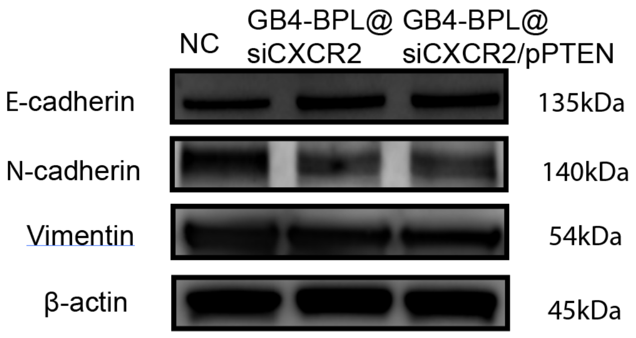

Figure S19. Protein expressions of E-cadherin, N-cadherin, and Vimentin were detected via western blotting in NC, GB4-BPL@siCXCR2, and GB4-BPL@siCXCR2/pPTEN groups (siCXCR2: 8.3 nM; pPTEN: 1.156 μg/mL).

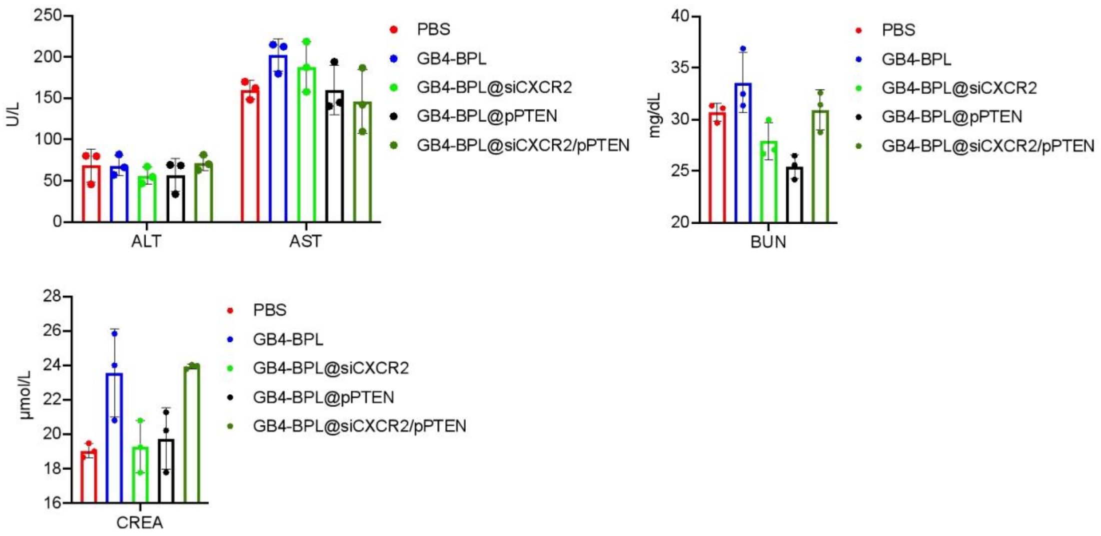

Figure S20. The biochemical analysis of serum alanine aminotransferase (ALT), aspartate aminotransferase (AST), blood urea nitrogen (BUN), creatinine (CREA) of mouse blood samples in BmCRPC mouse model (n = 3).Data are represented as means ± SD. **p* < 0.05, ***p* < 0.01, ****p* < 0.001, *****p* < 0.0001, ns: no significance, one-way ANOVA.

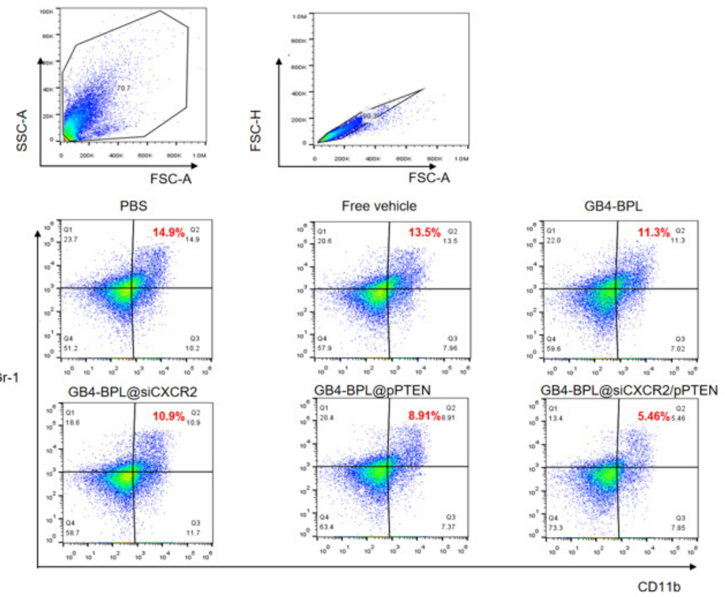

Figure S21. Gating strategy and typical flow cytometer analysis of MDSCs in tumors from each treatment. MDSCs were characterized as Gr-1^{+}CD11b^{+} cells.

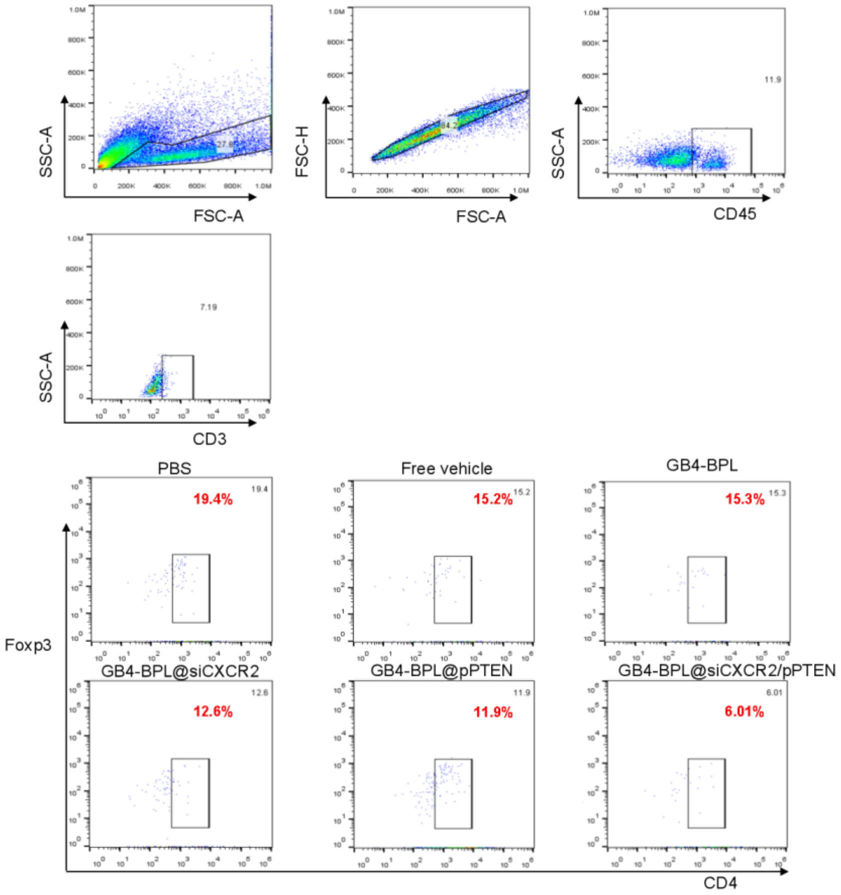

Figure S22. Gating strategy and typical flow cytometer analysis of Tregs in tumors

from each treatment. Tregs were characterized as $CD45^{+}CD3^{+}CD4^{+}Foxp3^{+}$ cells.

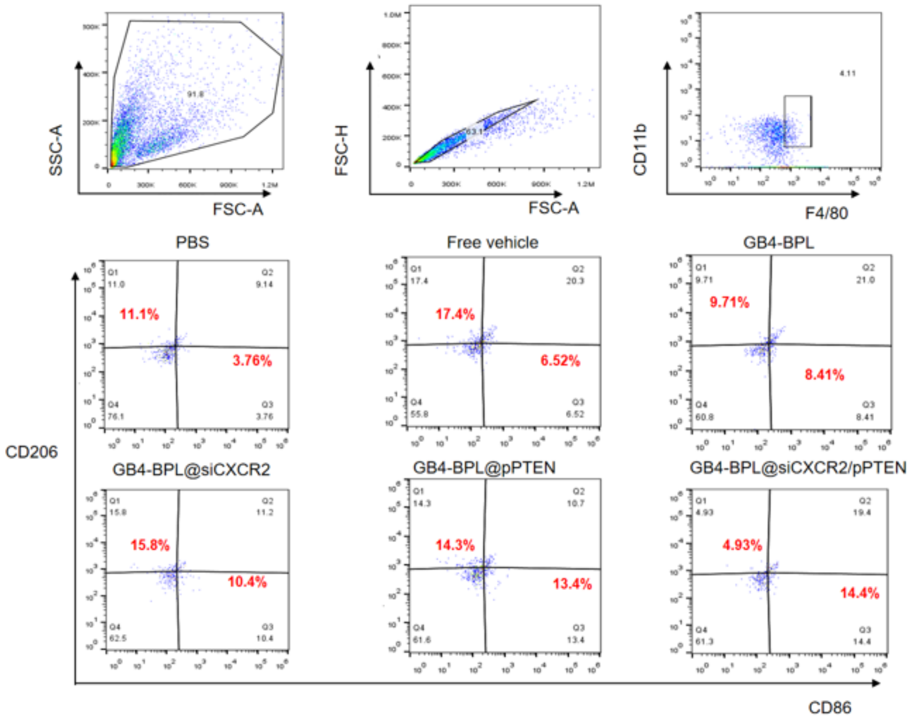

Figure S23. Gating strategy and typical flow cytometer analysis of M1-like or M2-like macrophages in tumors from each treatment. M1 macrophages were denoted as $CD11b^{+}F4/80^{+}CD86^{+}CD206^{-}$ cells while M2 macrophages were characterized as $CD11b^{+}F4/80^{+}CD206^{+}CD86^{-}$ cells.

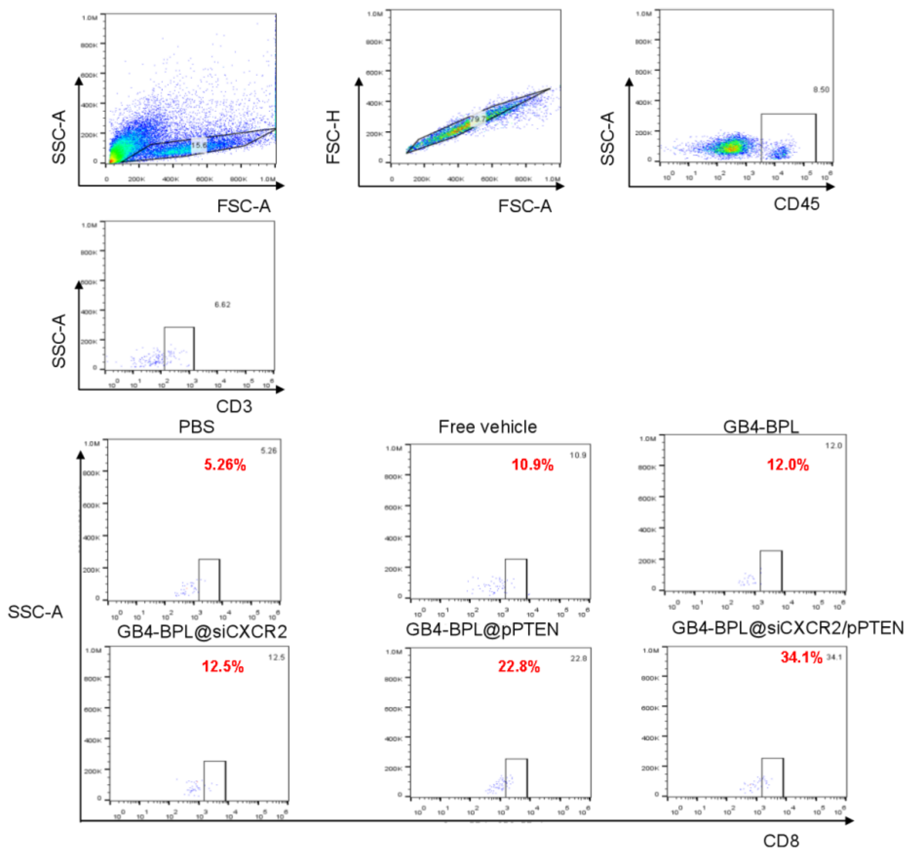

Figure S24. Gating strategy and typical flow cytometer analysis of $CD8^+$ T cells in tumors from each treatment. $CD8^+$ T cells were characterized as $CD45^+CD3^+CD8^+$ T cells.

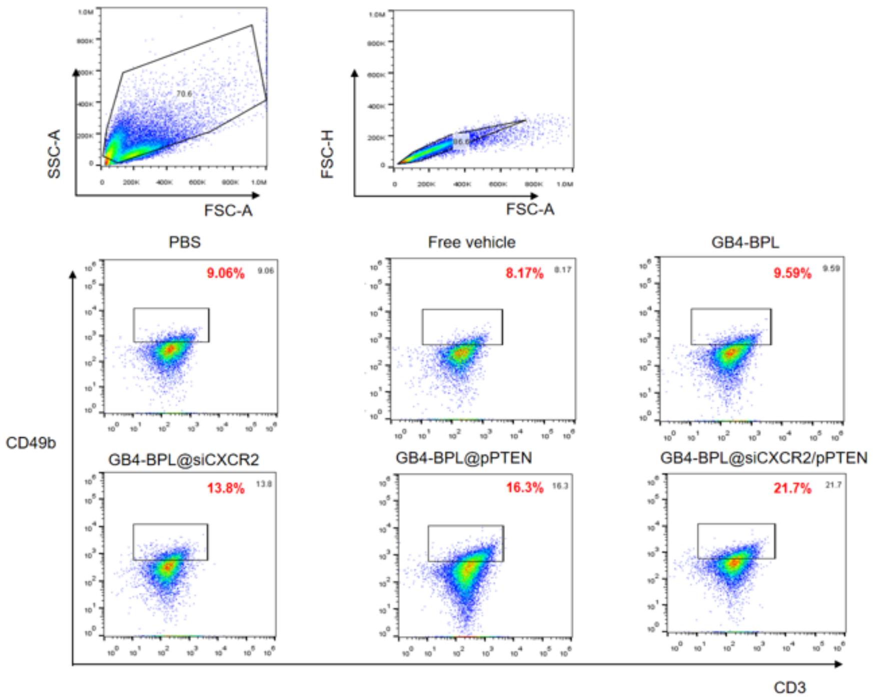

Figure S25. Gating strategy and typical flow cytometer analysis of NK cells in tumors from each treatment. NK cells were denoted as $CD3^-CD49b^+$ cells.
